# Lattice Strain Engineering on Metal‐Organic Frameworks by Ligand Doping to Boost the Electrocatalytic Biomass Valorization

**DOI:** 10.1002/advs.202403431

**Published:** 2024-06-03

**Authors:** Wenjing Bai, Xuan Wang, Jianing Xu, Yongzhuang Liu, Yuhan Lou, Xinyue Sun, Ao Zhou, Hao Li, Gengtao Fu, Shuo Dou, Haipeng Yu

**Affiliations:** ^1^ Key Laboratory of Bio‐Based Material Science and Technology of Ministry of Education Northeast Forestry University Harbin 150040 P. R. China; ^2^ Jiangsu Key Laboratory of New Power Batteries, Jiangsu Collaborative Innovation Center of Biomedical Functional Materials, School of Chemistry and Materials Science Nanjing Normal University Nanjing 210023 P. R. China; ^3^ Advanced Institute for Materials Research (WPI‐AIMR) Tohoku University Sendai 980–8577 Japan

**Keywords:** lattice strain, lignin depolymerization, MOFs electrocatalyst, β‐O‐4

## Abstract

As an efficient and environmental‐friendly strategy, electrocatalytic oxidation can realize biomass lignin valorization by cleaving its aryl ether bonds to produce value‐added chemicals. However, the complex and polymerized structure of lignin presents challenges in terms of reactant adsorption on the catalyst surface, which hinders further refinement. Herein, NiCo‐based metal‐organic frameworks (MOFs) are employed as the electrocatalyst to enhance the adsorption of reactant molecules through π‐π interaction. More importantly, lattice strain is introduced into the MOFs via curved ligand doping, which enables tuning of the d‐band center of metal active sites to align with the reaction intermediates, leading to stronger adsorption and higher electrocatalytic activity toward bond cleavage within lignin model compounds and native lignin. When 2′‐phenoxyacetophenone is utilized as the model compound, high yields of phenol (76.3%) and acetophenone (21.7%) are achieved, and the conversion rate of the reactants reaches 97%. Following pre‐oxidation of extracted poplar lignin, >10 kinds of phenolic compounds are received using the as‐designed MOFs electrocatalyst, providing ≈12.48% of the monomer, including guaiacol, vanillin, eugenol, etc., and p‐hydroxybenzoic acid dominates all the products. This work presents a promising and deliberately designed electrocatalyst for realizing lignin valorization, making significant strides for the sustainability of this biomass resource.

## Graphical abstract

By doping of isophthalic acid ligand into the NiCo‐based metal‐organic frameworks (MOFs), lattice strain is introduced. It successfully endows this MOFs with tunned electronic state of metal sites and facilitates electrocatalytic activities toward bond cleavage within the lignin model compounds and the pre‐oxidized poplar lignin under a low oxidation potential.

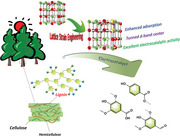

## 1 Introduction

The vigorous exploitation of the Earth's abundant and renewable biomass resources to produce value‐added chemicals and fuels is of great significance in alleviating the excessive consumption of conventional fossil resources.^[^ ^1^, ^2^, ^3^ ^]^ Depolymerization followed by the upgrading of natural biomass through catalytic reaction has attracted significant attention in both academic research and industrial manufacturing. Therein, electrocatalysis stands out as a highly effective approach for biomass upgrading due to its mild reaction conditions, environmental‐friendly nature, high conversion rates, and controllable reaction selectivity.^[^ ^4^, ^5^, ^6^, ^7^ ^]^ In electrocatalytic reactions, the adsorbing behavior of reactants and intermediates on the active sites is crucial for the overall process.^[^ ^8^ ^]^ Transition metal (Co, Ni, Cu)‐based materials, e.g., metal oxides, hydroxides, and derivatives, have shown promise as electrocatalysts for converting various biomass and the derived platform compounds.^[^ ^9^, ^10^, ^11^, ^12^, ^13^ ^]^ However, the most frequent transition metal‐based electrocatalysts usually show poor accessibility to biomass polymers, leading to a remained challenge of catalyzing depolymerization toward high product yields.^[^ ^14^ ^]^

On the other hand, as the most prevalent component biomass, lignin is the only natural polymer that contains abundant aromatic ring monomer structures. It is composed of three phenylalanine‐derived monomers, named p‐hydroxyphenyl, guaiacyl, and syringyl, connected by C─C/C─O bonds and β‐O‐4 moiety domains 40–60% of its composition.^[^ ^15^, ^16^, ^17^ ^]^ Efficient depolymerization of lignin through cleaving the C─C or C─O linkage can yield multifarious fragment products, particularly benzaldehyde derivatives such as vanillin, vanillic acid, syringic acid, and others. These derivatives have been used in fungicides, antioxidants, anti‐hardeners, drug intermediates, etc.^[^ ^18^, ^19^, ^20^, ^21^, ^22^ ^]^ Nevertheless, the intricate molecular structure and inherent degradation resistance of lignin still hinder its depolymerization toward high‐value utilization, especially through electrocatalytic process. Therefore, to break the depolymerization barrier for lignin valorization, enhancing the contact and adsorption between the electrocatalyst and reactant is of great importance.^[^ ^23^ ^]^

Metal‐organic frameworks (MOFs), formed by the coordination of organic ligands and metal nodes as periodic structural units with structural diversity and multifunctional tunable properties, provide a fertile possibility for optimizing the electronic structure of metal sites by tuning the first and/or second coordination shell, ultimately, obtaining high catalytic activities.^[^ ^24^, ^25^, ^26^ ^]^ More importantly, the organic ligands in MOFs, especially those with delocalization π‐electrons, such as phenyl‐based compounds, could offer non‐covalent interaction with reactants if lignin and its derivatives are employed as the reactants.^[^ ^27^, ^28^ ^]^ This property may promote steric adsorption between the catalyst and reactants, boosting the interaction between reactive sites and substrates during the reaction process.^[^ ^29^ ^]^ For another aspect, from the perspective of screening and material design, previous studies have shown that lattice strain could be introduced into MOFs through ligand modification to enhance the electrocatalytic oxygen evolution reaction performance.^[^ ^30^ ^]^ Under the lattice stress, the inherent interatomic distance changed, thereby regulating the bandgap and charge distribution, and optimizing the electronic structure of the active sites.^[^ ^24^ ^]^ In principle, this adjustment impacts the reactivity of metal sites by shifting the d‐band center.^[^ ^31^ ^]^ Tensile or compressive lattice strain can respectively strengthen or weaken the adsorption strengths of reaction species by altering the population of electrons in the anti‐bond energy band.^[^ ^31^, ^33^ ^]^ Consequently, tuning the d‐band center through inducing lattice strain by simple ligand modification not only optimizes the electronic structure of active sites but also fine‐tunes the interaction between adsorbate and the metal centers.

Herein, a NiCo‐based MOFs electrocatalyst with fabricated lattice strain was developed for catalyzing the cleavage of linkages in lignin and model compounds toward aromatic compounds. This MOFs structure was constructed by the coordination between terephthalic acid with Ni/Co metal ions. The benzene ring structure in the ligand provided enhanced adsorption with reactants through π‐π interaction. Simultaneously, lattice strain was introduced into the MOFs by doping with curved ligand, i.e., isophthalic acid (IPA). The altered coordination environment led to a strain effect in the MOFs and generated alternated d‐band center on the metal sites to boost the interaction with reactants and intermediates. When 2′‐phenoxyacetophenone was employed as the model compound, phenol, and acetophenone were obtained with yields of 76.3% and 21.7%, respectively, and a remarkable conversion rate of 97% was achieved. These results surpassed those obtained using pristine NiCo‐based MOFs and most previous reports in the literature. Moreover, when pre‐oxidized poplar lignin was used as the reactant, a noteworthy 12.48% of monomer products, including guaiacol, vanillin, and syringate, etc., were received. These findings demonstrate the efficiency of the as‐designed electrocatalyst in converting native lignin into value‐added monomers and highlighting its potential for lignin valorization and sustainable chemical synthesis.

## 2 Results and Discussion

**Figure 1:**
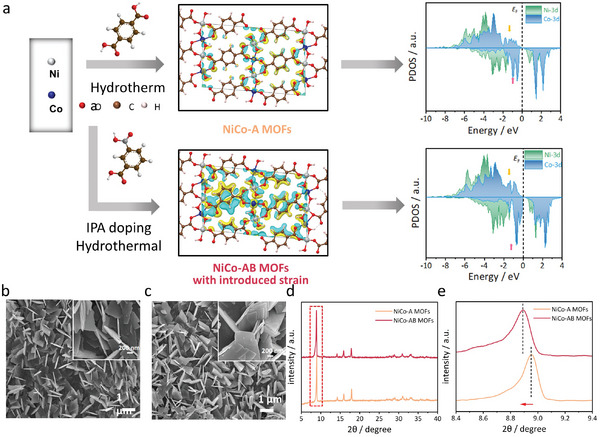
Structural characterizations of lattice‐strained MOFs. a) Illustration of the preparation of NiCo‐A MOFs and NiCo‐AB MOFs with the charge density difference on the two MOFs (yellow and blue colors represent charge accumulation and depletion, respectively), and the corresponding DOS of Ni and Co in NiCo‐A MOFs and NiCo‐AB MOFs; SEM of NiCo‐A MOFs; b) and NiCo‐AB MOFs; c) before and after ligand doping; d) XRD patterns of NiCo‐A MOFs and NiCo‐AB MOFs with e) amplified pattern.

The pristine NiCo MOFs (terephthalic acid as ligand, denoted as NiCo‐A MOFs) supported on carbon paper were prepared by a simple hydrothermal method, and the detailed process has been illustrated in the Supporting Information. For introducing strain in the NiCo‐A MOFs, curved carboxylic acid IPA was deliberately added, which could maintain the coordination tortuosity angle as a single variable, resulting in NiCo MOFs with topological changes (**Figure** **1**a), and the strain‐engineered MOFs were named as NiCo‐AB MOFs.

It is worth noting that, according to Figure S1 (Supporting Information), the energy gap between the highest occupied molecular orbital (HOMO) and the lowest unoccupied molecular orbital (LUMO) of IPA molecule is higher than the terephthalic acid, indicating its stronger ability to retain electrons, which could provide theoretical support for altering the electronic states of metal sites after coordination. Therefore, to probe the possible change of electronic structure after IPA doping, the charge density difference of two NiCo MOFs was first calculated. As shown in Figure 1a, after introducing curved ligands, NiCo‐AB MOFs exhibit significant charge localization around the molecule, and their spatial structural distribution also favors the local hydrogen bonding formation. To verify the electronic structure change of the material nodes related to the d‐band, DOS calculations were conducted. Partial density of states (PDOS) results show that ligand doping modulates the surface electronic structure of NiCo‐A MOFs. Specifically, the doping of IPA leads to lattice stretching, and the change in atomic spacing brings the Co‐3d state closer to the Fermi level, further promoting the electron transfer of Co as the main active site. As the average d‐band center of spin‐polarization shifts upwards, the d‐band center of metal atoms also migrates toward higher‐energy levels, resulting in a decrease in the amount of electron filling in the anti‐bonding energy band, thereby enhancing the adsorption interaction between reaction intermediates and substrates to improve the catalytic activity.^[^ ^34^ ^]^

The influence on the morphology of NiCo MOFs electrocatalysts after ligand doping was observed by scanning electron microscopy (SEM). Uniformly distributed 2D nanosheets NiCo‐A MOFs (Figure 1b) can be observed and the thickness of the sheets could be estimated as ≈40 nm. The thin layer structure would benefit from exposing more catalytic active sites, realizing the spatial separation of catalytic sites, and inhibiting their aggregation inactivation.^[^ ^33^, ^35^ ^]^ After strain introduction, the NiCo‐AB MOFs still maintain a similar morphology to the pristine (Figure 1c). However, by investigating the crystal structure of the MOFs before and after ligand doping, the strong diffraction peaks in Figure 1d of X‐ray diffraction (XRD) indicated that ligand doping did not change the diffraction pattern of the original MOFs, but the curved ligand caused the diffraction peak shift to a lower degree (from 8.95 to 8.89°, as shown in Figure 1e). This implies that the introduction of IPA ligand made the lattice to be stretched and increased the distance between the lattice planes, which is also direct evidence of successfully introducing strain in the MOFs.^[^ ^30^, ^34^ ^]^ The shift caused by lattice stress stretching further indicates the movement of the d‐band center, which would help boost the catalytic performance in subsequent electrochemical experiments. We also investigated the crystal structure of MOFs after different ligand doping levels. With the increase of IPA doping, the characteristic peak of MOFs shifted gradually  (Figure S2, Supporting Information), which well met the expectation. Moreover, the topological changes caused by ligand doping were further examined through nitrogen isothermal adsorption‐desorption (Table S1, Supporting Information) and thermogravimetric analysis (Figure S3, Supporting Information); results further implied that doping of the ligand could break the excellent periodic structure compared to the pristine MOFs, so that the NiCo‐AB MOFs owns higher porosity and pore volume than that of the NiCo‐A MOFs, and exhibiting a thermal solution shift of ≈9 °C. Therefore, it could be deduced that introducing IPA ligand partly changes the nanostructure of NiCo‐A MOFs, providing a more suitable environment for the interaction between the catalyst surface and biomass substrate. Additionally, even if a curved ligand was introduced, the molar ratio of Ni and Co remained ≈1:1 before and after doping, which was confirmed by the inductively coupled plasma mass spectrometry (ICP‐MS) quantification.

**Figure 2:**
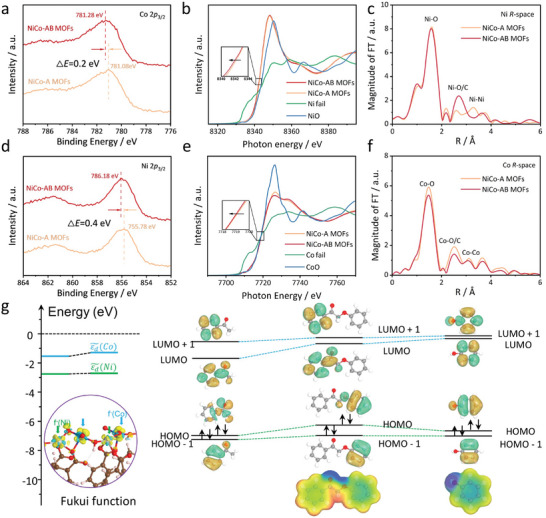
Electronic structures of NiCo MOFs before and after ligand doping. a) Ni 2p and b) Co 2p before and after ligand doping. b) Ni K‐edge and e) Co K‐edge XANES. c) Ni K‐edge and d) Co K‐edge FT curves of the EXAFS k^3^χ(k) functions. g) Schema of the relationship between molecular energy levels in different aggregated states.

To analyze the chemical composition and electronic structure of MOFs after lattice strain introduction, X‐ray photoelectron spectroscopy (XPS) was employed. **Figure** **2**a,d shows that both the binding energy of Co 2p_3/2_ and Ni 2p_3/2_ slightly shifted to higher binding energy (855.78 to 856.18 eV vs 781.08 to 781.28 eV) with the doping of IPA ligands, which is because the lattice strain reduces the electron density near metal atoms and modifies the local electronic structure. The valence band spectra (VBS) results in Figure S4 (Supporting Information) show that the valence band maximum of the NiCo‐AB MOFs shifted from the initial 0.68 to 0.48 eV. Obviously, after ligand doping, the valence band shifts toward the Fermi level. Since the valence electrons near the Fermi level mainly contribute to the d‐state, the movement of the valence band implies that the center of the d‐band in NiCo‐AB MOFs has also shifted under the effect of lattice strain compared to NiCo‐A MOFs. The upshift of the d‐band center favors enhanced adsorption and reactivity to the substrate, as confirmed by the results of the DOS. Figure 2b,e compares the X‐ray absorption near‐edge structure (XANES) of the metal sites in NiCo‐A MOFs and NiCo‐AB MOFs. Figure 2b indicates that the valence state of nickel is between nickel foil and nickel oxide, that is, a positively charged Ni^δ+^ species (0 < δ < 2), in contrast, the average valence state of cobalt in the catalyst is close to +2 (Figure 2e). After ligand doping, the absorption edge position of the metal slightly shifts to a lower energy level, indicating a change in the local electronic structure of the metal center under the stretching effect of stress. Figure 2c,f demonstrates the changes in the surrounding atomic structures using extended X‐ray absorption fine structure (EXAFS) spectroscopy, where the significant peak at 1.6 Å is assigned to the metal‐oxygen bond (M─O), and the relatively weak peaks at 2.5 and 3.1 Å are attributed to M─O/C and M─M bonds, respectively. In the *R* space, the intensity of EXAFS in NiCo‐A MOFs and NiCo‐AB MOFs differs mainly due to the changes in the local coordination geometry of the catalyst under the stress induced by ligand doping, leading to a transition from an originally ordered structure toward disorder. The change in the strength of electron interactions favors the improvement of subsequent catalytic performance. It has also been reported that the catalytic activity in MOFs structures lies in the unsaturated coordinating metal sites, and ligand doping could promote the generation of defect sites. To probe the most likely catalytic active site between the metal Ni and Co with the specific catalytic process, theoretical calculations were performed. As shown in Figure 2g, the frontier orbitals indicate that the HOMO and LUMO of the catalyst exhibit molecular polarization distribution. When interacting with the 2′‐phenoxyacetophenone molecule, which also exhibits charge polarization, the C─O bond of the model compound is able to be broken. Fukui functions also confirm the different roles of electron affinity of surface Co and Ni sites in the catalyst. After introducing the curved ligands, the exposed charge enrichment state is mainly in the 3d orbitals, which can symmetrically match with the LUMO of the lignin molecule. This enables continuous injection of electron states into the antibonding orbitals, thereby promoting the cleavage of ether bonds in the substrate owing to the favorable energy‐level match of the d‐band with frontier orbital levels.

**Figure 3:**
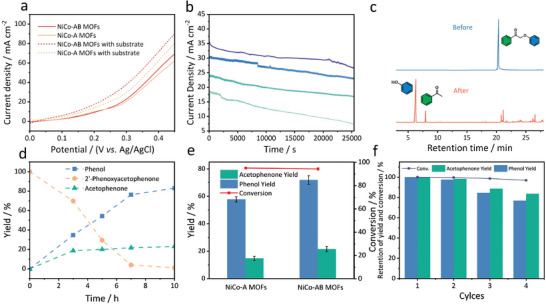
Influences of reaction parameters. a) Comparison of Linear Sweep Voltammetry (LSV) of NiCo MOFs before and after adding 2′‐phenylacetophenone (**1a**) in a mixed solvent system. b) IT curve of constant potential electrolysis of 2′‐phenoxyacetophenone (**1a**) at different potential gradients. From top to bottom, the potentials are 0.36, 0.34, 0.32, and 0.3 V (vs Ag/AgCl). c) Gas chromatography of 2′‐phenoxyacetophenone before and after depolymerization catalyzed by NiCo‐AB MOFs. Effect of d) reaction time and e) catalyst type on conversion rate and monomer yield of lignin model (2′‐Phenoxyacetophenone). f) Yield and conversion rate using NiCo‐AB MOFs in four continuous electrolysis.

As we have predicted, the development of MOFs‐based electrocatalysts is beneficial to improve the adsorption behavior between the reactants and catalyst through noncovalent interaction, i.e., π‐π interaction, a simple adsorption experiment was first carried out to investigate the adsorption capacity of NiCo MOFs after ligand doping. Compared to the NiCo(OH)_2_, as a reference sample, significantly higher current difference in the NiCo‐AB MOFs can be seen from Figure S5 (Supporting Information) at a potential higher than 0.2 V (vs Ag/AgCl), which implies the superior adsorption ability of lignin model compound on MOFs than the typical NiCo‐based hydroxide nanosheets. More importantly, considering the alternated d‐bond center induced from the strain engineering on electrocatalysts is probably to enhance the catalytic activity, which has been demonstrated on the small molecules conversion reaction, we believe that the as‐designed ligand‐doped MOFs may exhibit excellent catalytic performance on the lignin depolymerization.^[^ ^36^, ^37^, ^38^ ^]^ As well known that the cleavage of C─C/C─O bonds between aromatic monomers is the key step to realizing the lignin valorization, and noticed that plentiful studies have demonstrated that the lignin model compound with C_α_‐ketone could obviously weaken the bond dissociation energy in the β‐O‐4 structure than that with pristine C_α_‐alcohol. Therefore, to receive fundamental understanding of the relationship between the introduced strain effect and the electrocatalytic bond cleavage activity, dimer model compound 2′‐phenoxyacetophenone (**1a**) was employed as the reactant in our study throughout. The electrochemical reaction was carried out in an undivided electrolytic cell equipped with platinum (Pt) sheet as counter electrode, Ag/AgCl as reference electrode, and prepared catalyst NiCo‐AB MOFs as working electrode. Using organic solvents as electrolyte additives is an effective strategy for enhancing the solubility of complex substrates in potassium hydroxide.^[^ ^39^ ^]^ The activity of NiCo‐AB MOFs electrocatalyst was first investigated by linear scanning voltammetry (LSV); we can see from **Figure** **3**a that after adding 0.2 mmol **1a**, higher current density was obtained beyond 0.25 V versus Ag/AgCl, indicating that electrochemical oxidation reaction occurred obviously.^[^ ^40^ ^]^ In contrast, the current densities with **1a** on the pristine NiCo‐A MOFs were lower than that on the NiCo‐AB MOFs at any potential, verifying the enhanced catalytic activity by the strain introduction.

The catalytic bond cleavage performance on the strain‐engineered NiCo MOFs was evaluated under controlled potential electrolysis, and the products were qualitatively and quantitatively analyzed by gas chromatography‐mass spectrometry (GC‐MS) and gas chromatography (GC), respectively. To understand the relationship between the current density with the conversion rates and yields, electrolysis under different potentials (from 0.32 to 0.36 V vs Ag/AgCl) was first carried out to achieve desired current densities. As seen in Figure 3b, during the reaction, the current density gradually decreased, owing to the consumption of dimer reactants, phenol, and acetophenone as products accumulated in the electrolyte (Figure 3c). Note that the yield of acetophenone was lower than that of phenol, possibly because acetophenone is easy to be further fabricated to condensation. Since phenol dominated the products, the optimal current for lignin model compounds electrocatalytic cleavage was probed based on the phenol yield and **1a** conversion rate. By analyzing the GC results (Figure S6, Supporting Information), all conversion rates of **1a** at different potentials were >95%, indicating that electrochemical oxidation is efficient in cleaving the linkage of lignin model compounds. On the other hand, the highest phenol yield of 76.3% was received at the initial current density of ≈30 mA cm^−2^, at this moment, the corresponding applied potential was only 0.34 V versus Ag/AgCl. Furthermore, the optimal electrolysis time was also adjusted, and by prolonging the reaction time to 7 h (Figure 3d), substrate **1a** was almost consumed, and the conversion rate was calculated to be ≈97%. The obtained product yields of phenol and acetophenone gradually increased to 76.3% and 21.7%, respectively. To better understand the role of lattice strain in MOFs, the product yield on the electrocatalyst of NiCo‐A MOFs before ligand doping was also studied. As illustrated in Figure 3e, both the yields of phenol and acetophenone on the NiCo‐A MOFs were lower than that on the NiCo‐AB MOFs (i.e., 61.4% vs 76.3%, 15.8% vs 21.7%) at the same reaction conditions. These convincing evidences confirmed that the well‐designed electrocatalyst structure and the movement of the d‐band center provide more possibilities for realizing the depolymerization of lignin. Stability is a crucial factor in evaluating the performance of an electrocatalyst. Therefore, the durability of the lattice strain‐engineered NiCo‐AB MOFs was studied by several cycling electrolysis at 30 mA cm^−2^, and each cycle was maintained for 7 h. As shown in Figure 3f, the conversion rate for the **1a** well‐remained above 90%, and the product yield for phenol and acetophenone attenuated <20%, indicating that the MOFs‐based electrocatalyst processes excellent operational stability for the cleavage of lignin model compound. To explore the performance regulation of MOF by ligand doping with different bending angles, we also used phthalic acid with more significant bending angles to prepare lattice strained MOFs, NiCo‐AC MOFs (C represents phthalic acid) and NiCo‐A MOFs have the same diffraction pattern (Figure S7, Supporting Information). Compared with the pristine MOF, NiCo‐AC MOFs have a small low‐angle shift and show poorer crystallinity. The depolymerization results for 2′‐phenoxyacetophenone show that its catalytic ability is reduced. The angle of phthalic acid is too distorted, and the MOFs produced during the self‐assembly process have distortion, it may also have less doping amount, and the instability of the structure reduces its catalytic performance.

XRD results in Figure S8 (Supporting Information) demonstrate that after a long period of cycle stability testing, the catalyst did not transform into other substances. After 10 h of cyclic stability tests, characteristic diffraction peaks indicating MOFs crystallinity (≈8.9°) were still observable. However, after 35 h, no diffraction peaks were detected. The decrease in crystallinity of the catalyst may be due to surface passivation. At the same time, no XRD peaks of metal or metal oxide could be observed, indicating that no further transformation or reconstruction happened. On the other hand, the diffraction peak at 2θ = 26.5° corresponds to the graphitic carbon, which is due to the MOFs electrocatalyst being peeled from the carbon paper substrate and the inevitable introduction of carbon residual. Although SEM observations (Figure S9, Supporting Information) indicate the presence of passivation on the catalyst surface, TEM images (Figure S10, Supporting Information) still exhibit clear lamellar structures consistent with those before the cyclic tests (Figure S11, Supporting Information), suggesting that passivation may be limited to the catalyst surface. Concurrently, XPS results (Figure S12, Supporting Information) further imply limited changes in the chemical environment of the catalyst.^[^ ^25^ ^]^ All these results clearly confirmed that the structure of the as‐designed MOFs electrocatalyst does not change dramatically and it fits the mechanism of inference.

To study the substrate universality, we have prepared a series of model compounds with different spatial hindrances, which can help us analyze the catalytic bond‐cleavage activity of NiCo‐AB MOFs with simulated real lignin structure. Details of the substrate synthesis process are described in the Supporting Information. As shown in **Table** **1**, when 30 mA cm^−2^ was applied to electrolyze the selected compounds, corresponding aromatic ketones, and phenolic products were obtained with high reactant conversion rates of over 90%. When a methoxy group presents on the benzene ring connected to the ether bond, increased electron cloud density of the benzene ring due to the conjugation effect, causing the electron‐rich methoxyphenol to be prone to polymerization by the basic carrier, thus lower product yield obtained. Although the number of substituents did not further significantly affect the yield of phenolic products. When a para‐substituent on the aryl group connected to the carbonyl group, the impact on the yield of aromatic ketones is minimal. However, when both meta‐methoxy and para‐methoxy groups are present on the benzene ring, the yield of methoxy ketones decreases significantly. This is due to the increased spatial hindrance effect between the non‐planar conformation of the model compound and the electrocatalyst, which weakens the adsorption capacity on the catalyst surface. Simultaneously, the corresponding acetophenone products further condense during the electrooxidation process, resulting in generally lower ketone yields compared to phenolic compounds (Figure S13, Supporting Information). To get closer to the lignin structure, an electrocatalytic cleavage of a model compound containing γ‐OH was also applied (Table 1, entry 10), GC‐MS result confirmed that the products still contained only phenolic and ketone, which reveals that the presence of γ‐OH diminished the product yields, but did not affect the selectivity for bond cleavage. In short, through the exploration of different model compounds, it is confirmed that the NiCo MOFs after lattice strain engineering have high efficiency for cleaving the bonds in β‐O‐4 moiety.

**Table 1:** Oxidative cleavage range of a typical lignin model.

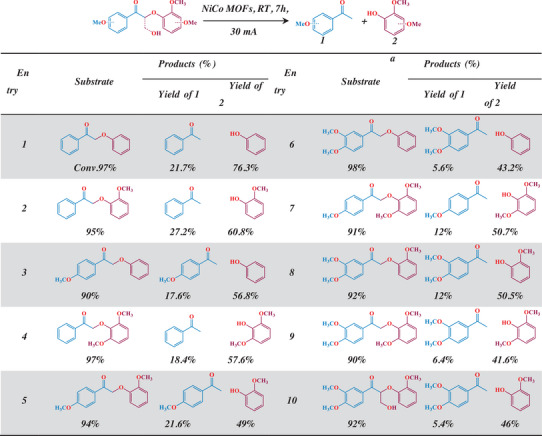

Conditions: Substrate (0.2 mmol), EtOH (2.5 mL), THF (2.5 mL), 1 M KOH (5 mL), 30 mA cm^−2^, 7 h at room temperature, unless otherwise noted.

**Figure 4:**
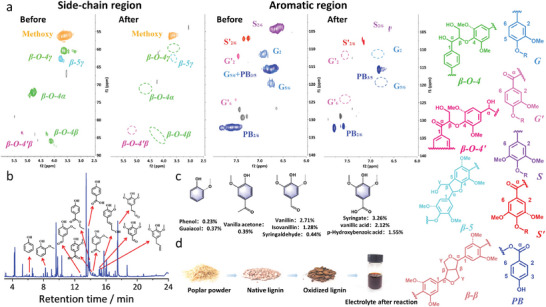
a) Partial 2D HSQC NMR spectra of poplar lignin before and after the electrocatalytic reactions. b) GC chromatograms of electrocatalytic depolymerization of lignin. c) Main fragment products of electrocatalytic depolymerization of lignin. d) Pictures of poplar wood powder, native lignin, oxidized lignin, and the electrolyte after the reaction.

To lower the bond dissociation energy within the β‐O‐4 moiety in native lignin, pre‐oxidation of poplar lignin with DDQ has been conducted to obtain a benzyl ketone‐containing structure.^[^ ^41^ ^]^ The results showed that DDQ has good reactivity and selective conversion of α‐OH in lignin as demonstrated in Figure S14 (Supporting Information). The 2D HSQC NMR spectra of poplar lignin (typical hardwood lignin) extracted by organic solvent method before and after DDQ pre‐oxidation exhibited significantly overlapping ^13^C‐^1^H crossover signals with significant group changes belonging to their α, β, and γ‐ moieties, respectively.^[^ ^42^ ^]^ The β‐O‐4 content is 41% among all the aromatic units, and clear signal presence in the aromatic region means that both S and G type lignin monomers were partially oxidized to their benzyl ketone analogs S' and G'. For example, weakened β‐O‐4 α (δ_C_ 73, δ_H_ 5.0 ppm) signal and clarified β‐O‐4′ signal (δ_C_ 83, δ_H_ 5.7 ppm) indicates that the C_α_−OH bond was oxidized to C═O and the signal changes occurring in the aliphatic region are matched. Considering the excellent universal applicability to β‐O‐4 model compounds, we conducted a preliminary attempt on depolymerization of real lignin by electrocatalysis. 70 mg of pre‐oxidized poplar lignin was dissolved in a mixed solvent for 10 h continuous electrolysis under the optimal conditions of model exploration, that is, 0.34 V versus Ag/AgCl (Figure S15, Supporting Information). By measuring the 2D nuclear magnetic resonance (NMR) of the lignin residue after the reaction (**Figure** **4**a), it was observed that the signals for β‐O‐4 and β‐O‐4′ in the side chain region disappeared, while the signal for methoxy groups weakened, along with a reduction in the signals for syringyl (S) and guaiacyl (G) units in the aromatic region. These observations suggest that ether bonds may have partially cleaved during the electrocatalytic depolymerization process, indicating the catalyst effectively cleaves specific bonds within the lignin structure and leading to the production with smaller, more valuable chemical components. Thirteen products in the extracted solvent were detected by GC chromatograms (Figure 4b–d), including vanillin, syringic acid, parahydroxybenzoic acid, and acetovanillone, etc., of which syringic acid, p‐hydroxybenzoic acid, and vanillin were the main products, accounting for 3.26%, 1.55%, and 2.71%, respectively, and the total monomer yield was 12.48% (based on the initial weight of lignin). In contrast, only vanillin and allyl eugenol (Figure S16, Supporting Information) in the unoxidized poplar lignin were obtained as the products, the inferior monomer yield is due to the higher dissociation energy barrier to cleave the linkage in β‐O‐4 with C_α_−OH. It is also worth noting that when native lignin was applied as the reactant, the selectivity of products was still poor. This may be related to the complex lignin structure and complicated electrochemical mechanism during the reaction, including some spontaneous hydrolysis processes. This issue is also the bottleneck within the field of lignin refinement, which will be the focal point of our future research.

## 3 Conclusion

In this study, we successfully introduced lattice strain into NiCo‐based MOFs through the doping of isophthalic acid ligands, optimizing the electron density near the metal atoms and the antibonding states above the Fermi level. This strain‐induced modulation of the electronic structure caused the d‐band center of the active sites to align with the reaction intermediates, leading to stronger adsorption capacity and higher catalytic activity. As a result, efficient electrocatalytic transformation of lignin model compounds and lignin was achieved. Using a constant potential of only 0.34 V versus, the NiCo‐AB MOFs material exhibited excellent bond‐breaking performance for β‐O‐4 dimers and native lignin. The yield of monomers obtained from model compounds is superior to or comparable to most related studies on the electrochemical depolymerization of lignin model compounds. This provides a new pathway for the stable conversion of biomass lignin into value‐added chemicals. In future studies, in addition to pre‐oxidation, the introduction of specific functional groups into lignin fragments can further enhance the utilization of biomass for product value upgrading or increase product yields closer to theoretical values under electrocatalysis.^[^ ^43^ ^]^

## Conflict of Interest

The authors declare no conflict of interest.

## Supporting information

Supporting Information

## Data Availability

The data that support the findings of this study are available from the corresponding author upon reasonable request.
