# Supplementary material for: Lattice Strain Engineering on Metal‐Organic Frameworks by Ligand Doping to Boost the Electrocatalytic Biomass Valorization
Source: Adv Sci (Weinh). 2024 Jun 3;11(29):2403431. doi: 10.1002/advs.202403431 (PMC11304310; doi:10.1002/advs.202403431)
Supplement: Supplementary file 1 — Supporting Information [file ADVS-11-2403431-s001.docx]

**Supporting Information**

Lattice Strain Engineering on Metal-Organic Frameworks by Ligand Doping to Boost the Electrocatalytic Biomass Valorization

Wenjing Bai,^#^ Xuan Wang,^#^ Jianing Xu, Yongzhuang Liu, Yuhan Lou, Xinyue Sun, Ao Zhou, Hao Li, Gengtao Fu*, Shuo Dou*, and Haipeng Yu*

W. Bai, J. Xu, Y. Liu, Y. Lou, X. Sun, A. Zhou, S. Shuo, H. Yu

Key Laboratory of Bio-based Material Science and Technology of Ministry of Education, Northeast Forestry University, Harbin, 150040 (P. R. China)

E-mail: doushuo@nefu.edu.cn, yuhaipeng20000@nefu.edu.cn

X. Wang, G. Fu

Jiangsu Key Laboratory of New Power Batteries, Jiangsu Collaborative Innovation Center of Biomedical Functional Materials, School of Chemistry and Materials Science, Nanjing Normal University, Nanjing, 210023 (P. R. China)

E-mail: gengtaofu@njnu.edu.cn

H. Li

Advanced Institute for Materials Research (WPI-AIMR), Tohoku University, Sendai, 980-8577 Japan

^#^ These authors contributed equally to this work.

**Experimental section**

1. Catalysts preparation and characterization

**Material** Cobalt (II) nitrate hexahydrate (98%), Nickel (II) chloride hexahydrate, terephthalic acid (99%), isophthalic acid (99%), phthalic acid (99%), N, N′-dimethylformamide (DMF), ethanol, and were purchased from Aladdin (Shanghai, China). Solvents were purchased from commercial sources. All the chemicals were used without any further purification.

**Preparation of NiCo-A MOFs (NiCo BDC)** Cobalt nitrate hexahydrate (0.8 mmol) and nickel chloride hexahydrate (0.8 mmol) were dissolved in a mixture of 60 mL DMF, 2 mL ethanol, 2 mL of water, and 1.6 mmol of terephthalic acid to obtain a homogeneous solution, followed by transferring to a 100 mL Teflon-lined autoclave placed with hydrophilic carbon paper (2 cm×5 cm). Hydrothermal treatment was carried out at 120 ^o^C for 12 h, and after the reactor naturally cooled to room temperature, the carbon paper loaded with NiCo-A MOFs was washed with water and ethanol and dried at room temperature. The mass loading of NiCo-A MOFs on carbon paper was ~2 mg/cm^2^.

**Preparation of NiCo-AB MOFs** Cobalt nitrate hexahydrate (0.8 mmol) and nickel chloride hexahydrate (0.8 mmol) were dissolved in a mixture of 60 mL DMF, 2 mL ethanol, and 2 mL of water. Terephthalic acid (1.44 mmol) and phthalic acid (0.16 mmol) or isophthalic acid (0.16 mmol) were then added into the above solution to obtain a homogeneous solution and transferred to a 100 mL Teflon-lined autoclave where hydrophilic carbon paper (2 cm×5 cm) was placed at the bottom. After 12 h hydrothermal treatment at 120 ^o^C, the carbon paper loaded with NiCo MOFs was washed with water and ethanol, and dried at room temperature. Regarding the doping of the curved ligands, we uniformly chose 10 mol%. The mass loadings of NiCo-AB MOFs and NiCo-AC MOFs on carbon paper were also ~2 mg/cm^2^, respectively. (where A stands for terephthalic acid, B stands for isophthalic acid, and C represents phthalic acid)

**Electrochemical measurements.** Electrochemical measurements were performed in a three-electrode system controlled by a CHI 760E workstation. The Ag/AgCl and platinum plate electrode were used as the reference and counter electrode, respectively. The prepared NiCo MOFs on the carbon paper were directly used as working electrode. LSV curves were recorded at a scan rate of 0.1 mV/s. The potential in the LSV polarization curves were corrected by iR compensation at 90%.

**General procedure for electrochemically cleavage of dimer models.** 0.2 mmol dimer substrate was dissolved in the mixed electrolyte, after a short period of sonification and stirring to obtain a homogeneously dispersed solution, constant potentials were applied and the entire reaction process was continuous. All the electrolyte in the cell was transferred to a glass bottle, extracted using an equal amount of ethyl acetate. Saturated NaCl solution was added during the extraction process, and water drying was performed using anhydrous magnesium sulfate. After filtered by a 0.22 μm organic filter, 1 mL of product solution was transferred to an injection bottle to perform the qualitative and quantitative analysis with gas chromatography-mass spectrometry (GC-MS, Agilent 7890A GC-5975C) and gas chromatography (GC, Agilent 8860), respectively. Chemicals in product mixtures were identified by GC-MS: The heating program is 50 ^o^C for 3 min, rises to 100 ^o^C at a rate of 25 ^o^C/min for 3 min, and rises to 300 ^o^C at a rate of 15 ^o^C/min for 3 min. The products were quantified using a GC with a flame ionization detector (FID) and HP-5MS column.

**Characterizations.** X-ray diffraction (XRD) data were recorded on a Rigaku Smartlab 3KW diffractometer with a Cu-Kα X-ray radiation source (λ = 1.54056 Å). The sample morphology was photographed using a TESCAN MIRA LMS scanning electron microscope. The carbon paper (loaded catalyst) was fixed directly to the conductive glue, and Quorum SC7620 sputter coater was used to spray gold for 45 s, spray gold at 10 mA. The X-ray photoelectron spectroscopy (XPS) was conducted by the ThermoFisher scientific Kα XPS (XPS spectra were referenced to the C 1s peak of 284.8 eV). Co/Ni K-edge X-ray absorption fine structure (XAFS) analysis were performed with Si (111) crystal monochromators at the BL14W1 beamlines at the Shanghai Synchrotron Radiation Facility (SSRF) (Shanghai, China). Before the analysis at the beamline, samples were pressed into thin sheets with 1 cm in diameter and sealed using Kapton tape film. The XAFS spectra were recorded at room temperature using a 4-channel Silicon Drift Detector (SDD) Bruker 5040. Extended X-ray absorption fine structure (EXAFS) spectra of Co/Ni were recorded in transmission mode. Negligible changes in the line shape and peak position of Co and Ni K-edge XANES spectra were observed between two scans taken for a specific sample. The XAFS spectra of standard samples (CoO and Co fail, NiO and Ni fail) were also recorded in transmission mode. The spectra were processed and analyzed by the software codes Athena and Artemis.

**2. Synthesis of Lignin β-O-4 Model Compounds^1^**

**Material:** Phenol, guaiacol, 2,6-dimethoxyphenol, 2-bromoacetophenone, 4-methoxy-α-bromoacetophenone, bromo-3,4-dimethoxyacetophenone, alpha-Bromo-4-Hydroxyacetophenone, acetone, potassium carbonate, diatomaceous earth, and ethanol were obtained from Aladdin. All the chemicals were used without any further purification. (2-Phenoxyacetophenone purchased from Aladdin, 1-(3,4-dimethoxyphenyl)-2-(2-methoxyphenoxy)-3-hydroxy-1-propanone purchased from APINNO)


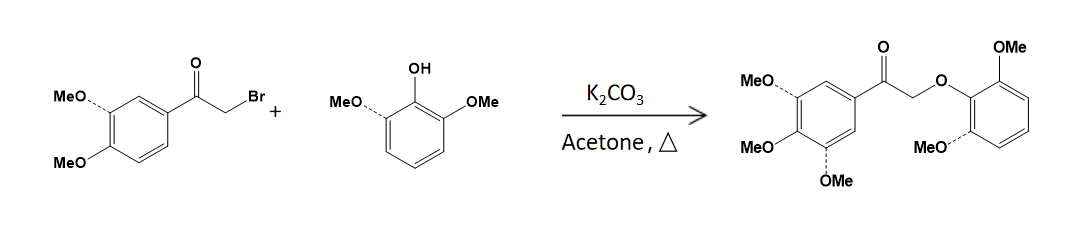


**General procedure for preparation of 2-Phenoxyacetophenone (1b-1i):**

The substrates used in this work were synthesized in two steps from the corresponding phenolic chemicals (phenol, guaiacol, 2,6-dimethoxyphenol) and bromoacetophenone chemicals (2-bromoacetophenone, 4-methoxy-α-bromoacetophenone, bromo-3,4-dimethoxyacetophenone). First, the bromoacetophenone chemicals (5 mmol) were added to potassium carbonate (7.5 mmol) in 50 mL of acetone, and then the phenolic chemicals (6.25 mmol), also dissolved in 50 mL of acetone, were slowly dropped into the prepared solution. The mixed solution was stirred at the reflux temperature for 5h, then filtered out with diatomaceous earth under vacuum and concentrated by spin-steaming. It was then dissolved in 20 mL of ethanol solution for recrystallization to obtain white or yellowish crystals. The NMR data of all products were consistent with those reported in the literature.

**3. Preparation of oxidized birch lignin**

**Extraction of lignin:** Before extraction, poplar wood was ground into powder to increase the area of solid-liquid contact. Poplar sawdust was then placed in a filter paper packet and placed in an extractor which was connected to a round-bottom flask containing the leaching solvent with a return condenser. The leaching solution was a mixed solution of 400 mL of 1,4-dioxane with 1.6 mL of hydrochloric acid and 12 mL of deionized water. After cooling, the filtrate was filtered and concentrated by vacuum. After the addition of 1 L of water and 10 g of ammonium chloride to the concentrate with quickly stirring, poplar lignin could be collected by filtration and dry at room temperature.

**DDQ pre-oxidized lignin**^2^**:** The organosolv lignin obtained in the previous stage was dissolved in 7 mL of 1,4-dioxane, followed by the addition of 2,3-dichloro-5,6-dicyano-1,4-benzoquinone (10 wt%) and NaNO_2_ (20 wt%). The mixture was stirred under an O_2_ atmosphere at 80 ^o^C. After 5h, precipitate and filter in excess deionized water were conducted to obtain pre-oxidized lignin. The color of pre-oxidized poplar lignin changed to brown, indicating the α-OH in the β-O-4 unit was pre-oxidized to Cα=O.

**2D HSQC NMR analyses**^3^: 2D heteronuclear single quantum coherence nuclear magnetic resonance (2D HSQC NMR) spectra were recorded on a Bruker AVANCE HD 500 MHz spectrometer at room temperature. The organosolv lignin before and after pre-oxidation, as well as after electrolysis were recorded by dissolving the corresponding samples (60-90 mg) in 0.7 mL DMSO-d6 (when incompletely soluble in d6-DMSO, adding a few drops of D_2_O). The solvent peak was used as an internal reference standard (δ_C_ 39.5, δ_H_ 2.49 ppm). The HSQC experiment had a spectral width of 20 ppm for F2 (^1^H dimension), with 2048 data points and an acquisition time of 128 ms. The F1 (^13^C dimension) had a spectral width of 219 ppm, with 512 increments (11.6 ms) and a delay of 1.5s (D1) between each of the 20 scans.

**Quantitative analysis of lignin depolymerization products:** 70 mg of pre-oxidation lignin was used as substrate for electrocatalytic depolymerization, and the lignin was firstly dispersed in the mixed electrolyte by ultrasonic and stirring, and the concentration of the product increased with prolonging electrolysis reaction time. The quantitative analysis of the lignin depolymerization products after the reaction was carried out by the external standard method. Before that, the reaction solution was transferred to a glass bottle, and acidified by dropping sulfuric acid to pH=2~3, the remaining lignin could be precipitated, Then, ethyl acetate was used for extraction, and anhydrous magnesium sulfate was used as dehydrating agent. After filtering by 0.22 μm organic filter, the chemicals in the mixture were identified by GC-MS. An autosampler was used to inject about 1.0 µL sample in a split mode (split ratio 60:1). The injection port is 300 ^o^C, the initial temperature is 50 ^o^C and kept for 2 minutes, using rate of 25 ^o^C/min to increase to 100 ^o^C. After that, the rate of 10 ^o^C /min was applied to increase the temperature to 300 ^o^C, and maintaining for 1 minute.

1. Density functional theory (DFT) calculations

First-principle calculations were operated *via* the Vienna *ab initio* Simulation Package (VASP) with spin-polarization.^4,5,6^ The projector-augmented-wave (PAW) method was used to describe the core electrons.^7,8^ For the description of electronic exchange and correlation, the revised Perdew-Burke-Ernzerhof functional (RPBE) was used as developed by Nørskov and colleagues.^9^ Valence electrons were treated by expanding the Kohn-Sham wavefunctions in a plane-wave basis set with the energy cutoff of 500 eV. A Gamma-centered *k*-point Brillouin zone sampling was used, with the grid density of 3×3×1. To model the active surface, we choose the (001) surface for the exposure of metal coordinatively undersaturated sites (CUS), which are commonly assumed as the active sites. The geometric optimization was allowed with a force convergence threshold of 0.03 eV/Å. For electronic self-consistent field (SCF) calculation, a convergence threshold of 10^-5^ was applied. To avoid the periodic interaction, we added the 15 Å vacuum layer along the z direction for the slab model. For quantum chemistry calculations, we chose the Gaussian 16 package to investigate the molecular properties.^10^ The electronic structure computations were performed at B3LYP level with the basis set of 6-311G*.^11,12^

HOMO and LOMO structures of 2'-phenoxyacetophenone before and after bond breaking. The iso-surface value is 0.02 e/Å^3^.


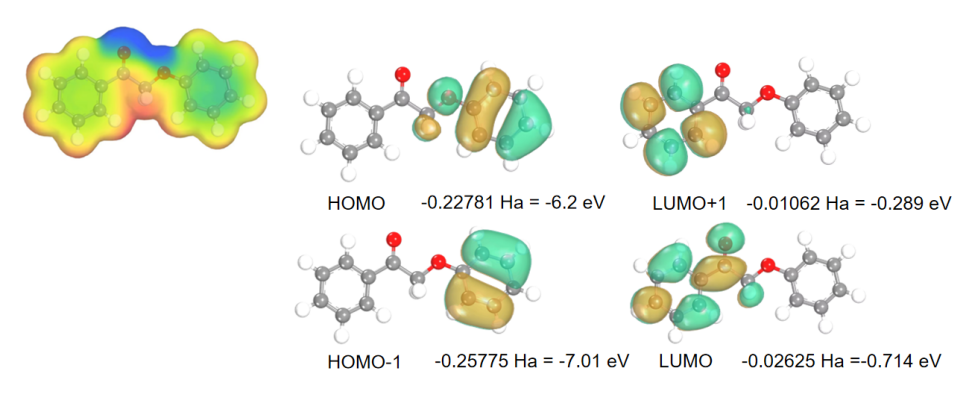


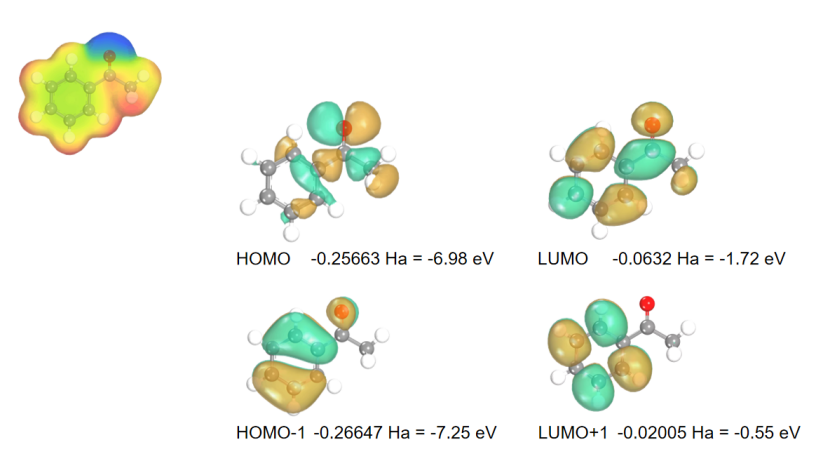


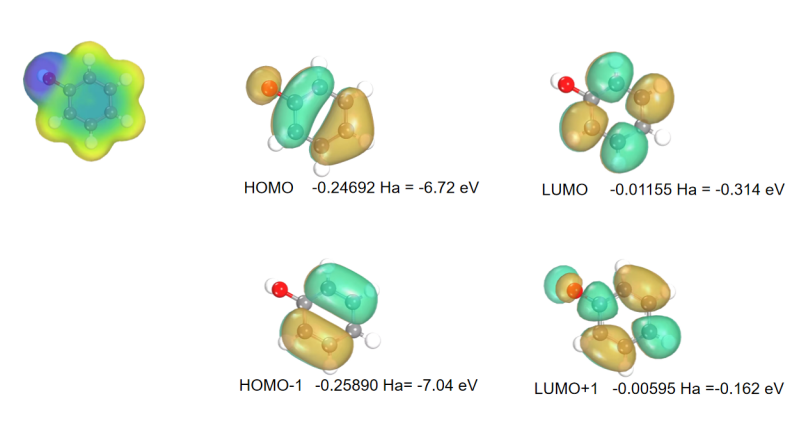


^1^H NMR Spectra of Lignin β-O-4 Model Compounds


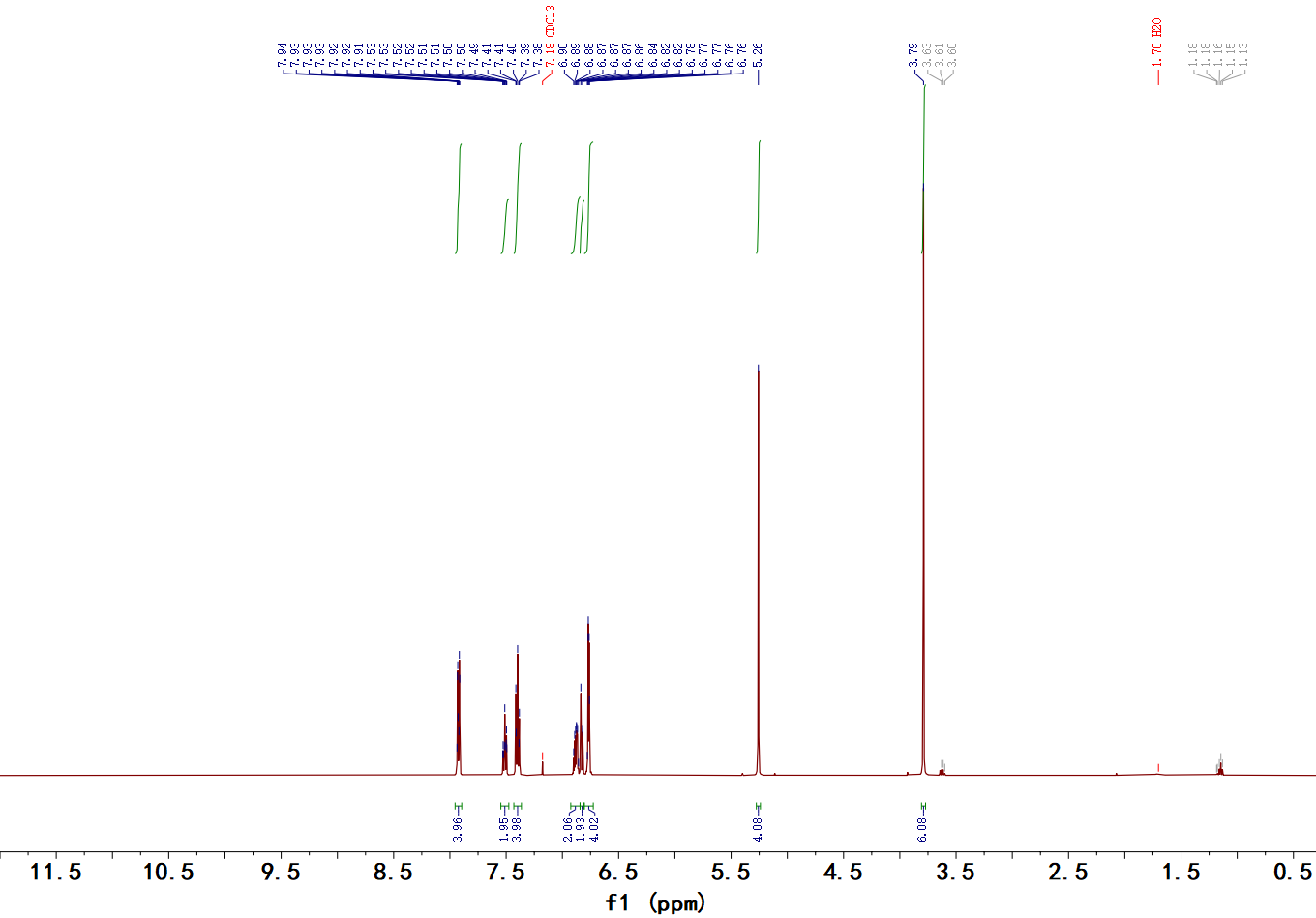


1b


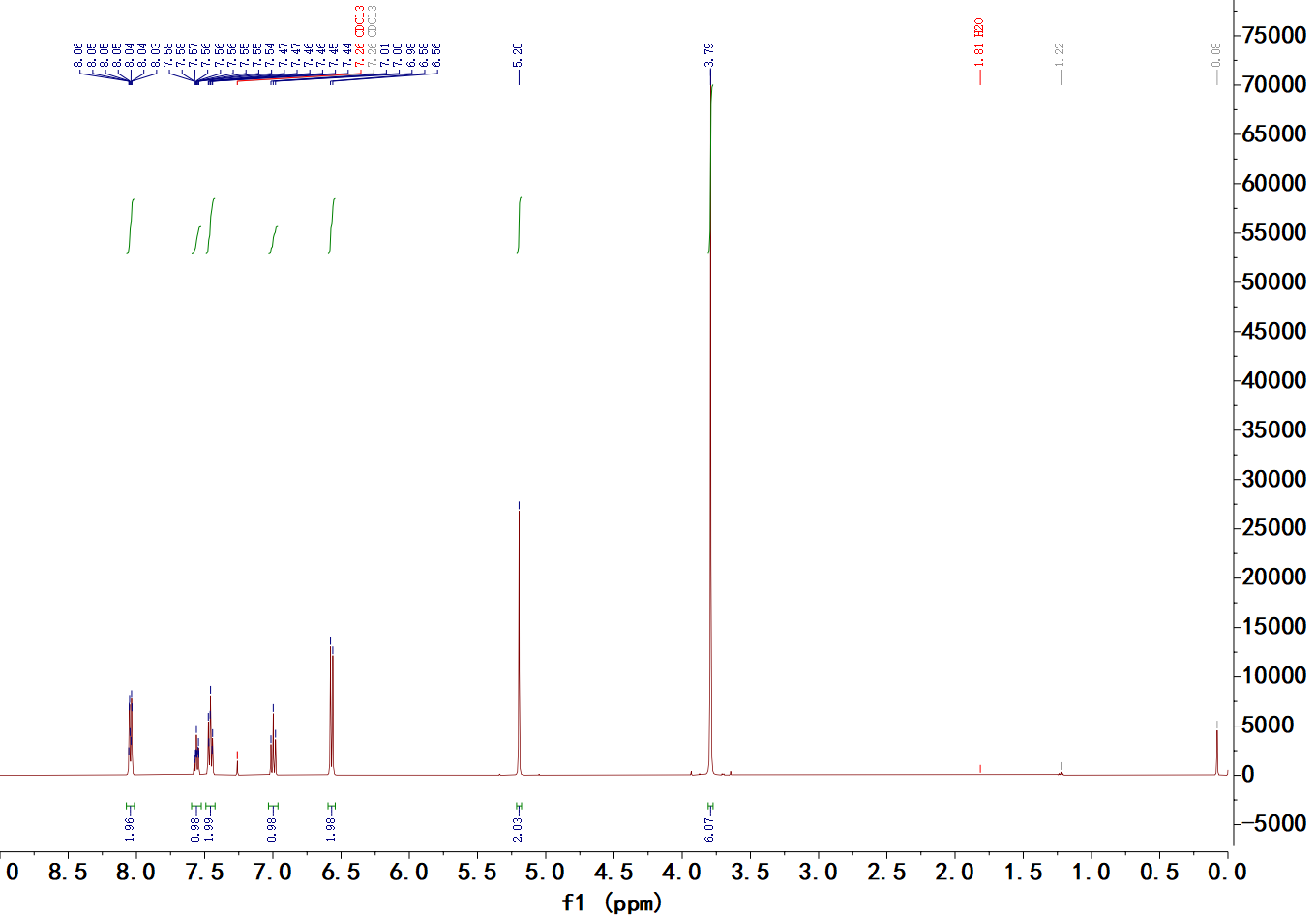


1c


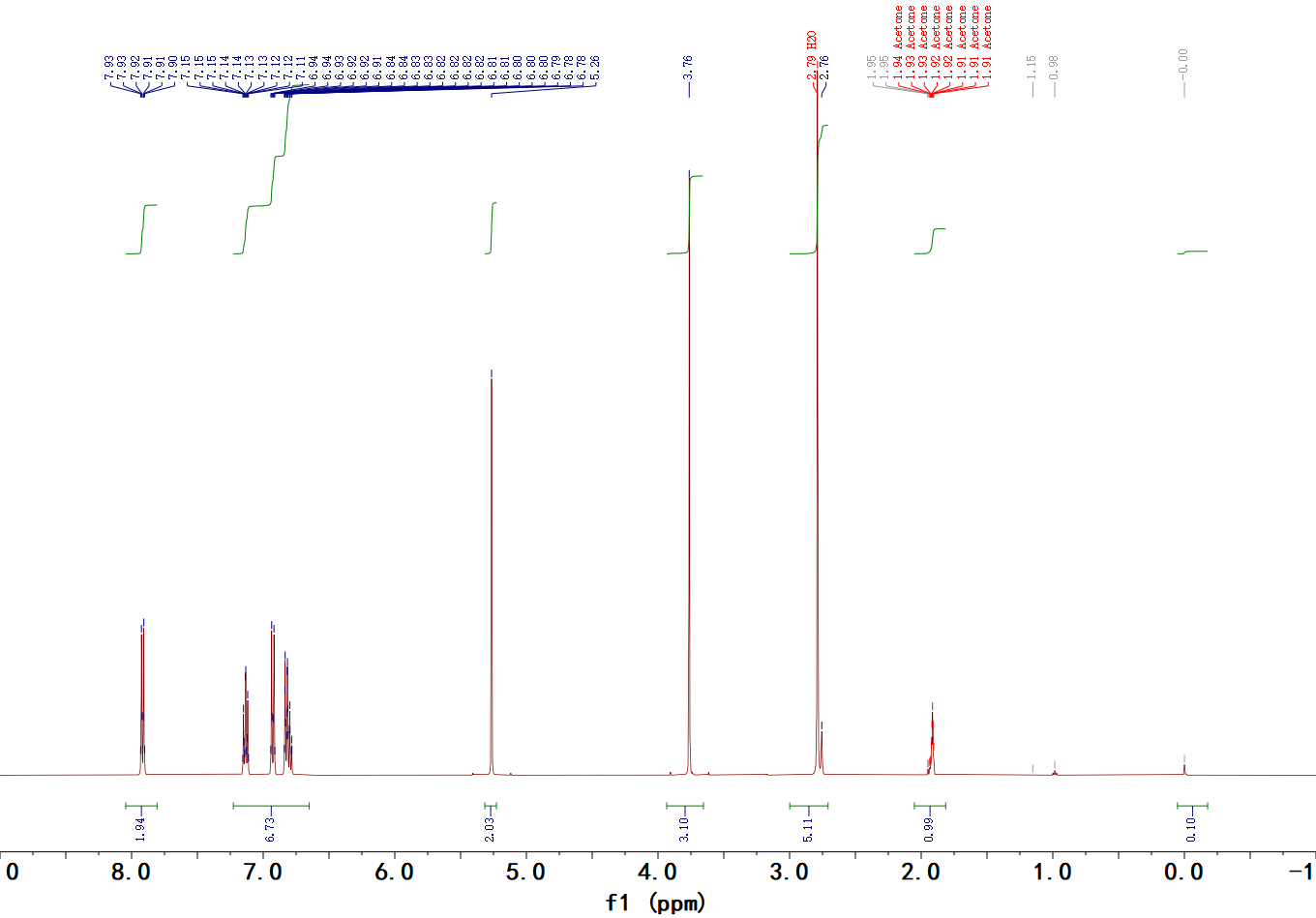


1d


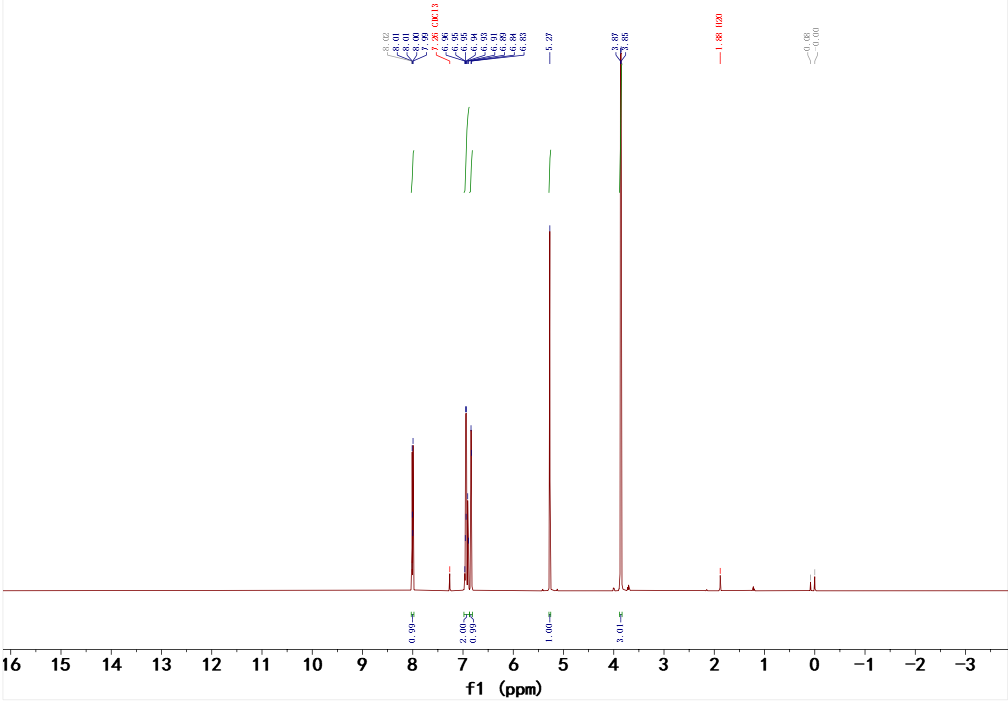

1e


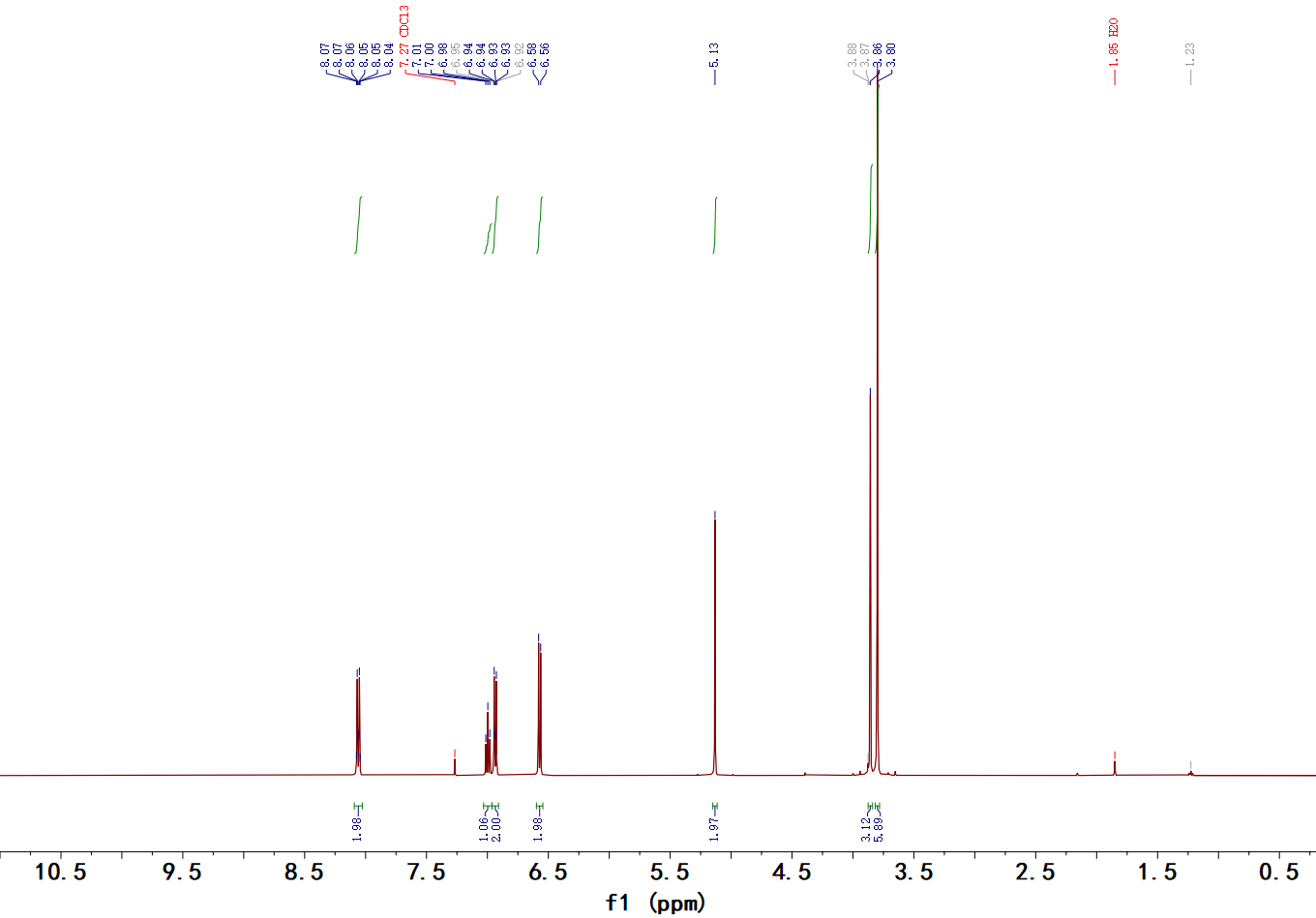


1f


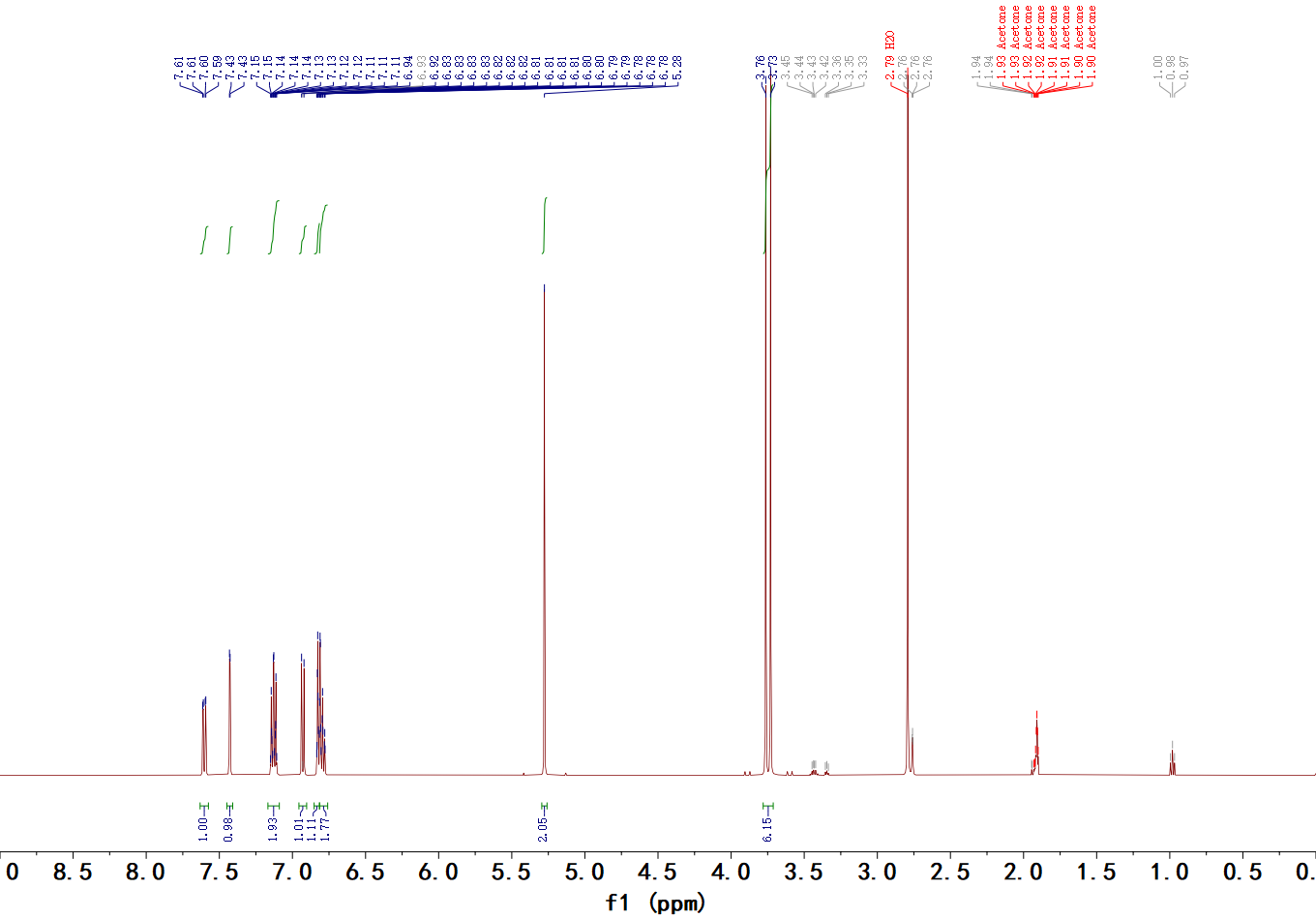


1g

Standard curve of the conversion rate of dimer substrate versus product yield of depolymerized substrate.


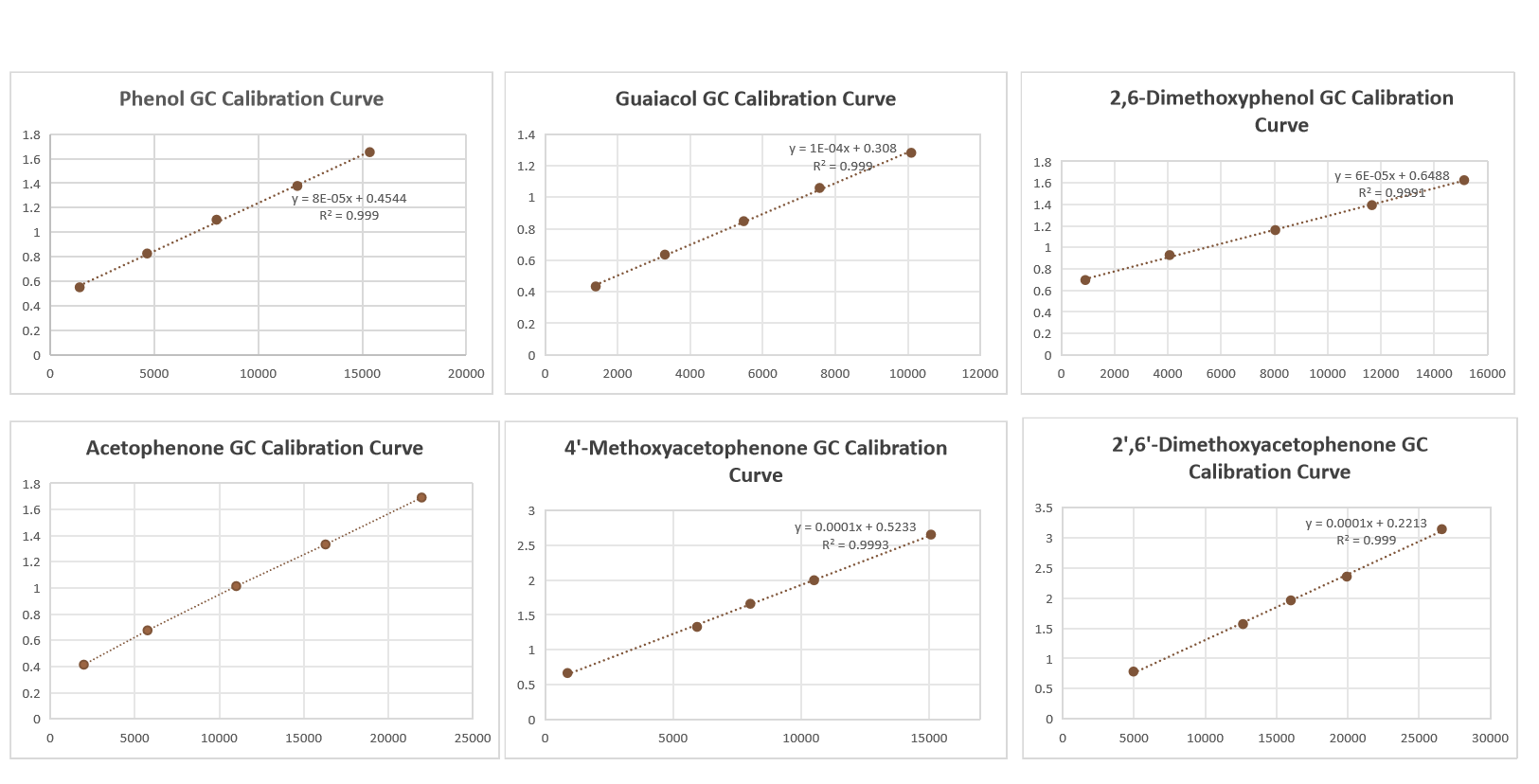


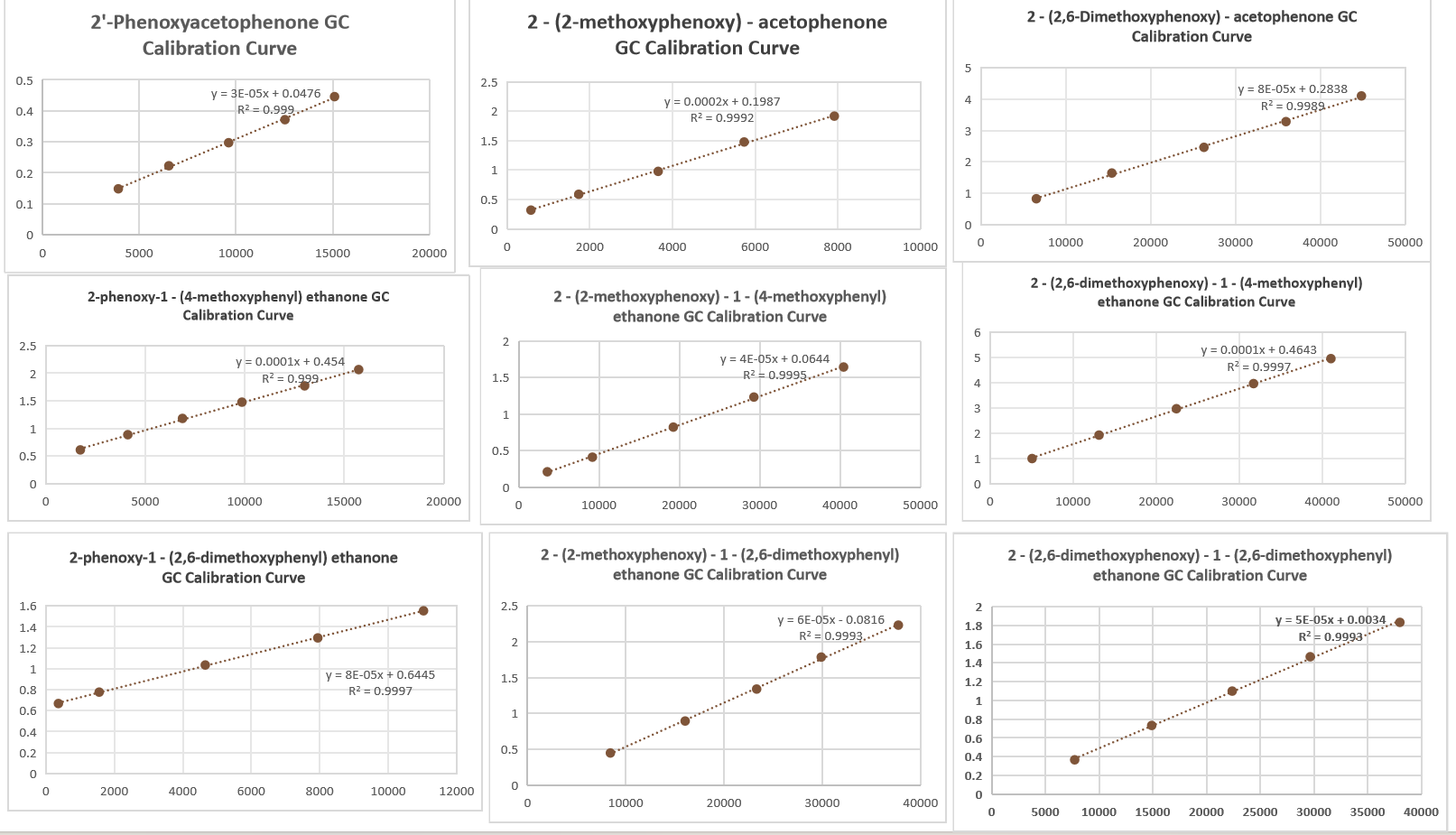


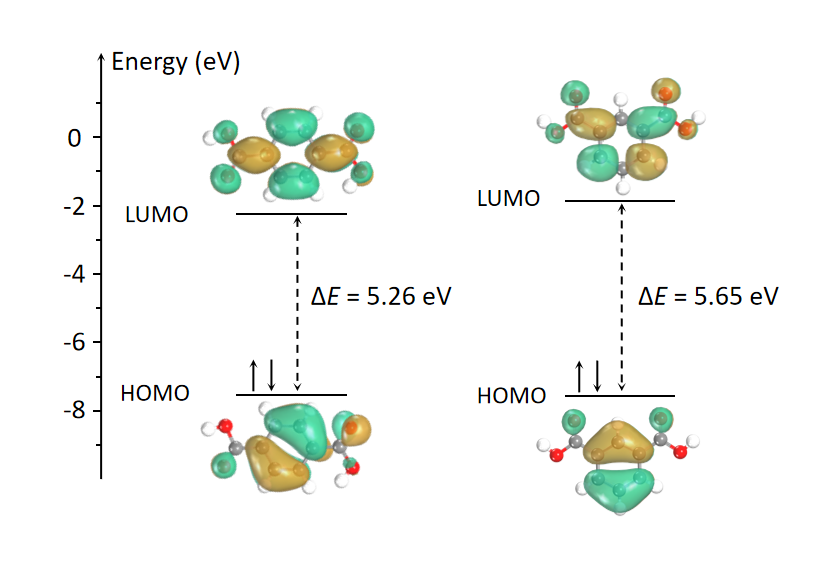


**Figure S1.** **Gap values of HOMO-LUMO in different ligand molecules with the iso-surface value of 0.02 e/Å^3^**.


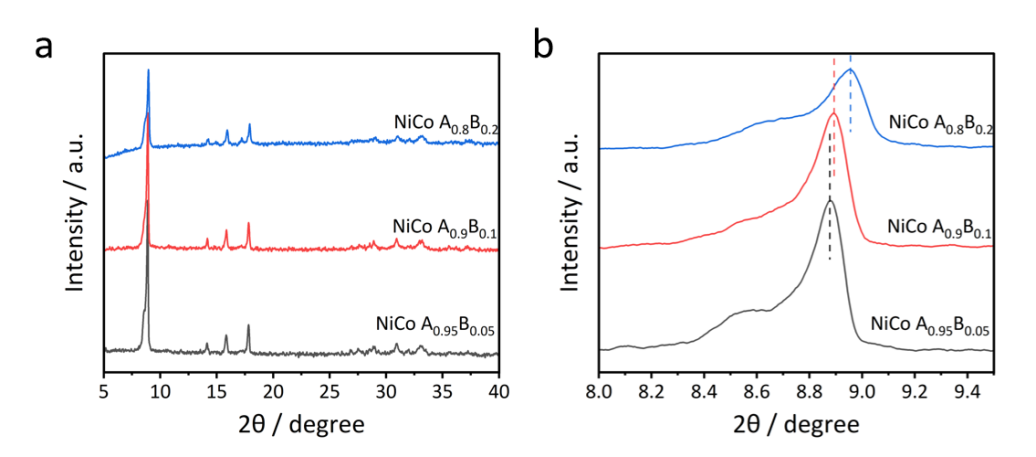


**Figure S2. XRD patterns of 5%, 10%, 20% isophthalic acid doped MOFs**. In order to highlight the doping ratio more intuitively, the naming convention of NiCo A_x_B_y_ was chosen, where NiCo A_0.9_B_0.1_ is NiCo-AB MOFs, and x and y represent the doping ratio of terephthalic acid and isophthalic acid. With the increase of isophthalic acid doping content, the characteristic peak of MOF shifts gradually.^13^


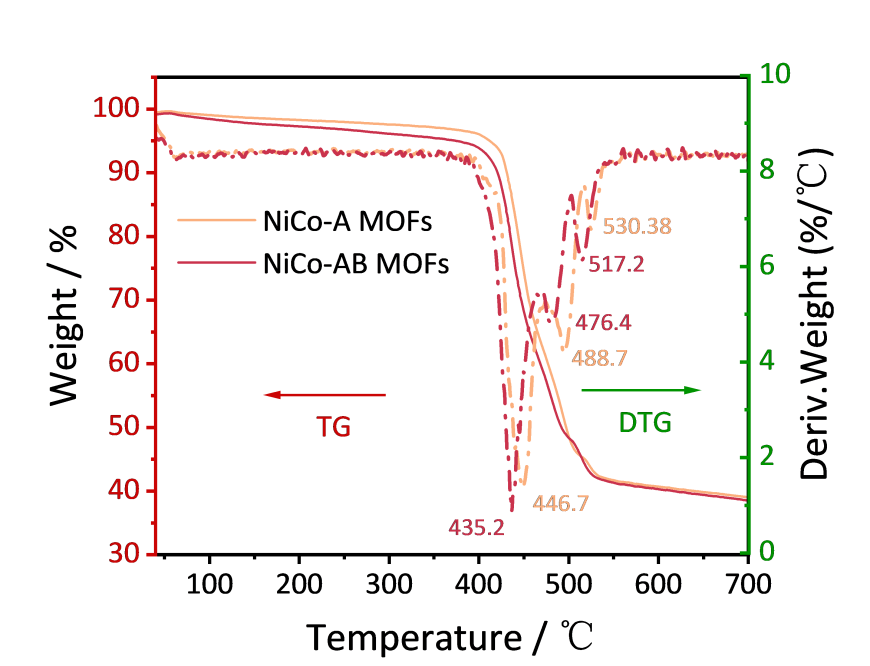


**Figure S3. TGA before and after ligand doping**: The anterior section of the weight loss curve is gentle, losing water at about 100~120 ^o^C, and beginning to change abruptly at about 400 ^o^C, indicating that the sample begins thermal degradation. Due to ligand doping, defects were introduced to NiCo-AB MOFs, and more unstable structures exhibited pyrolysis shifts of about 9 ^o^C.


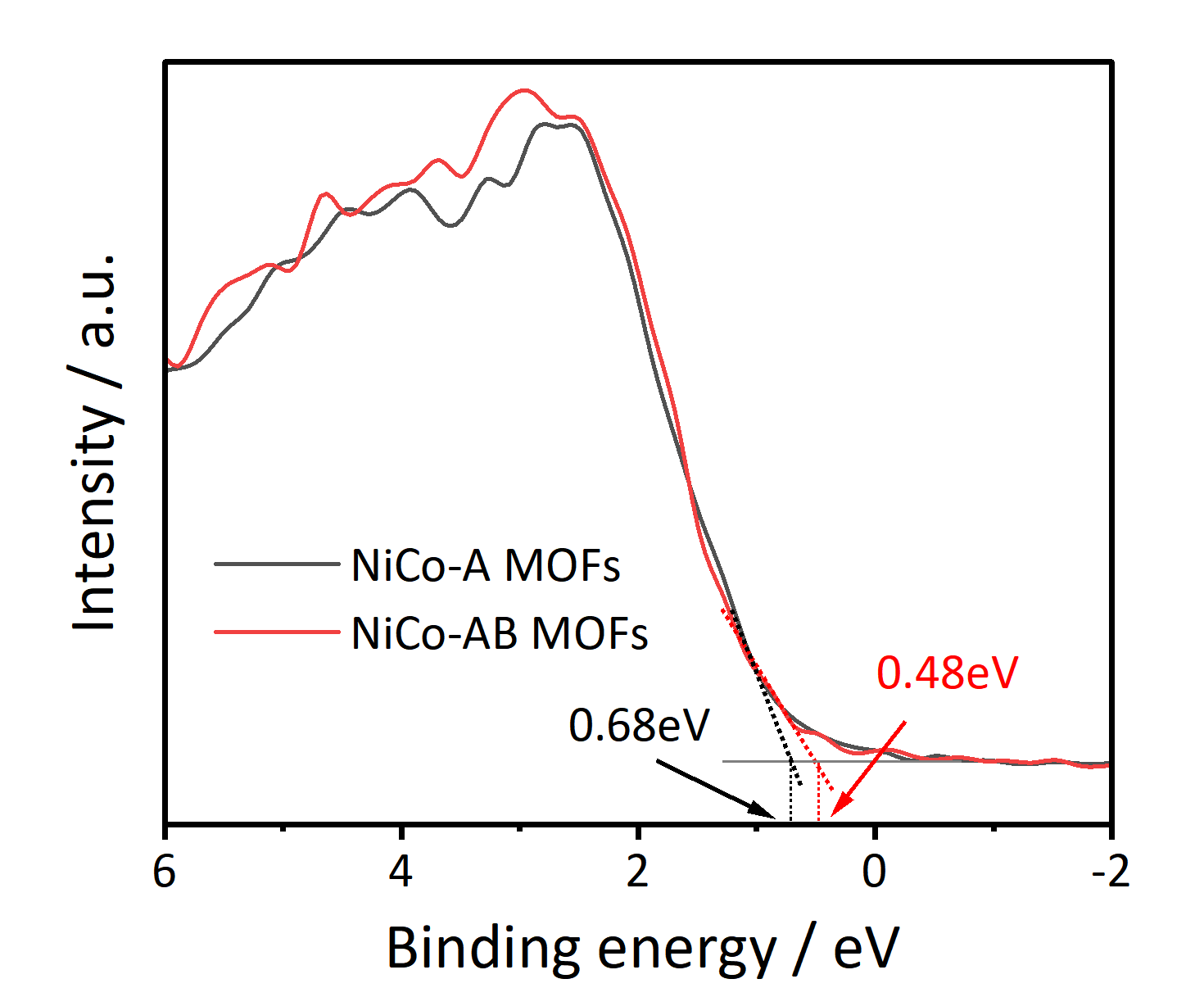


**Figure S4. Valence band spectroscopy (VB-XPS) results before and after ligand doping**.


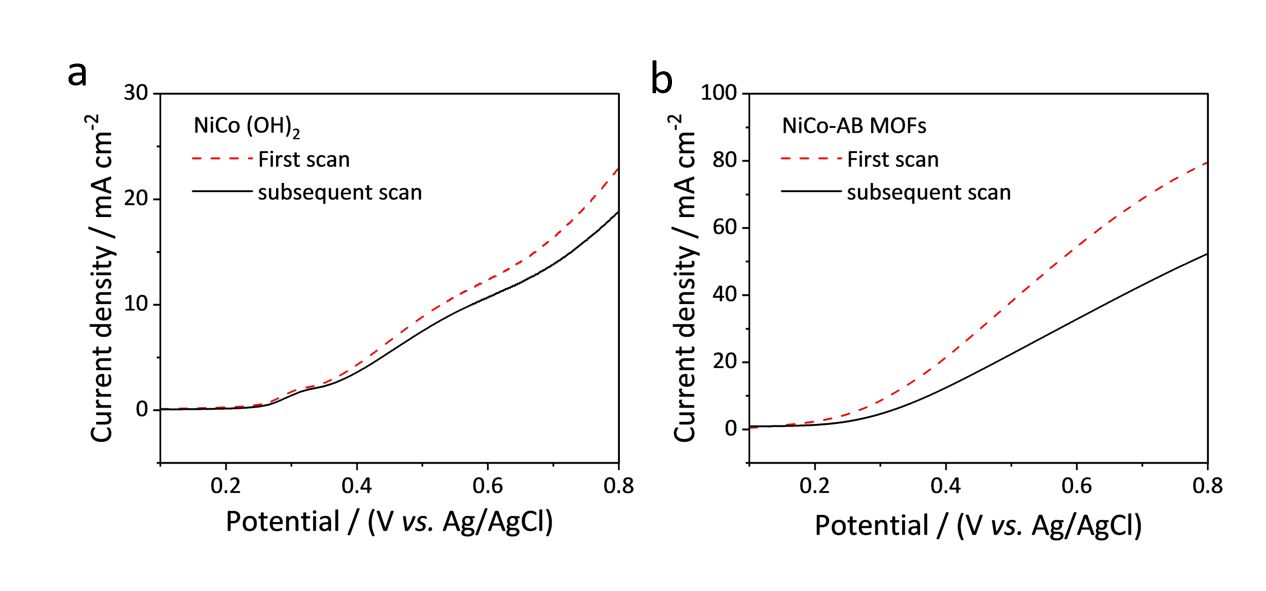


**Figure S5. Adsorbate stripping from 2'-Phenoxyacetophenone adsorption on(a) NiCo-AB MOFs and (b) NiCo(OH)_2_ catalysts^14^.** Reaction conditions: 1M KOH/THF/EtOH=2:1:1; scan rate = 10 mV s^−1^. First scan (dash red line) and subsequent scans (solid lines).

For electrochemical adsorption peel measurements, NiCo-AB MOFs and NiCo(OH)_2_ samples (on hydrophilic carbon paper) were first immersed in a mixed solution containing 0.3 mM 2'-Phenoxyacetophenone and scanned at 0.3 V (*vs.* Ag/AgCl) for 1 min to ensure substrate adsorption, where an appropriate potential (sufficient to adsorb the substrate but not catalytic the oxidized substrate bond breaking) needs to be selected to ensure the normal continuation of the subsequent reaction. After adsorption, excess model matter needs to be removed by rinsing the working electrode in O_2_-free ultrapure water, and then transferred to an electrochemical electrolytic cell containing a mixed solvent to electrocatalyze the adsorbed 2'-phenoxyacetophenone by LSV scanning. Although the complex solvent system obscured the oxidation peak, making it impossible to calculate Q_Ox_ (2'-Phenoxyacetophenone), the results of LSV still showed that NiCo-AB MOFs adsorbed more substrate.


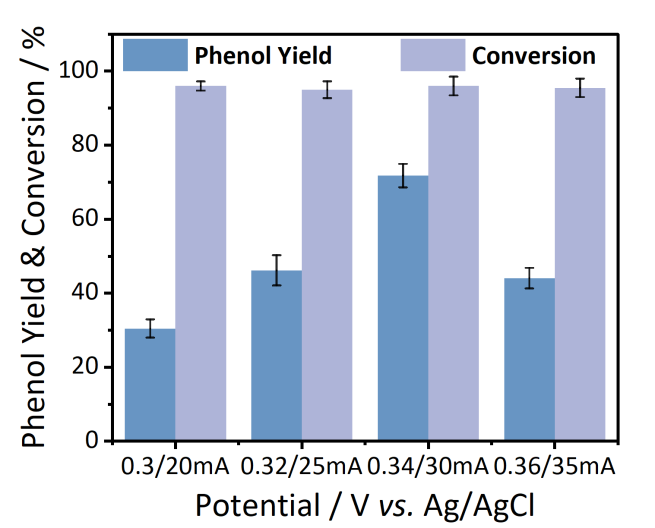


**Figure S6. The optimal reaction potential (current density) based on product yield and conversion rate.**


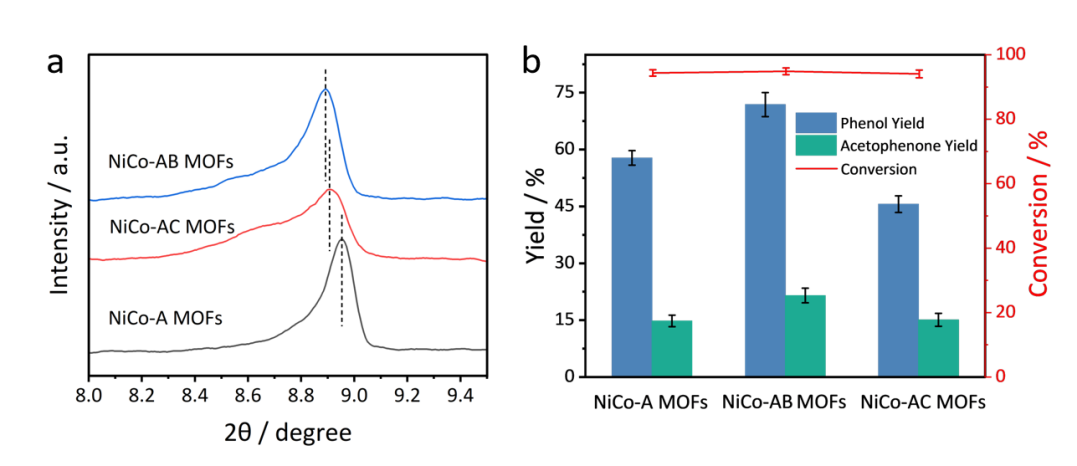
**Figure S7. (a) XRD patterns of NiCo-A MOFs, NiCo-AB MOFs, and NiCo-AC MOFs; (b) Conversion rate and product yield under different electrocatalysts.**


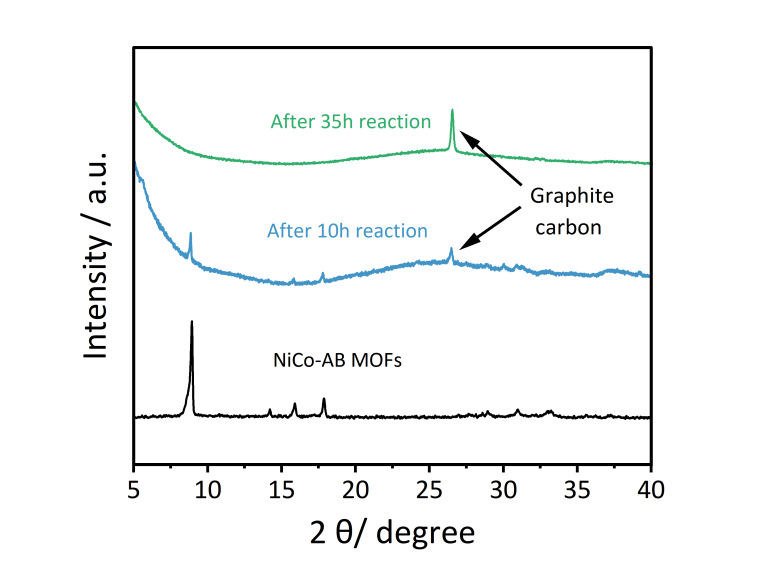


**Figure S8. XRD patterns of NiCo-AB MOFs before and after cycles test.**The diffraction peak at 2θ=26.5°corresponds to the graphitic carbon from the carbon paper substrate of the catalyst. During the scraping process of the catalyst, a portion of the carbon paper loaded with the catalyst also fell off. Since the catalyst before the reaction was directly prepared by hydrothermal synthesis, there were no corresponding graphitic carbon diffraction peaks.


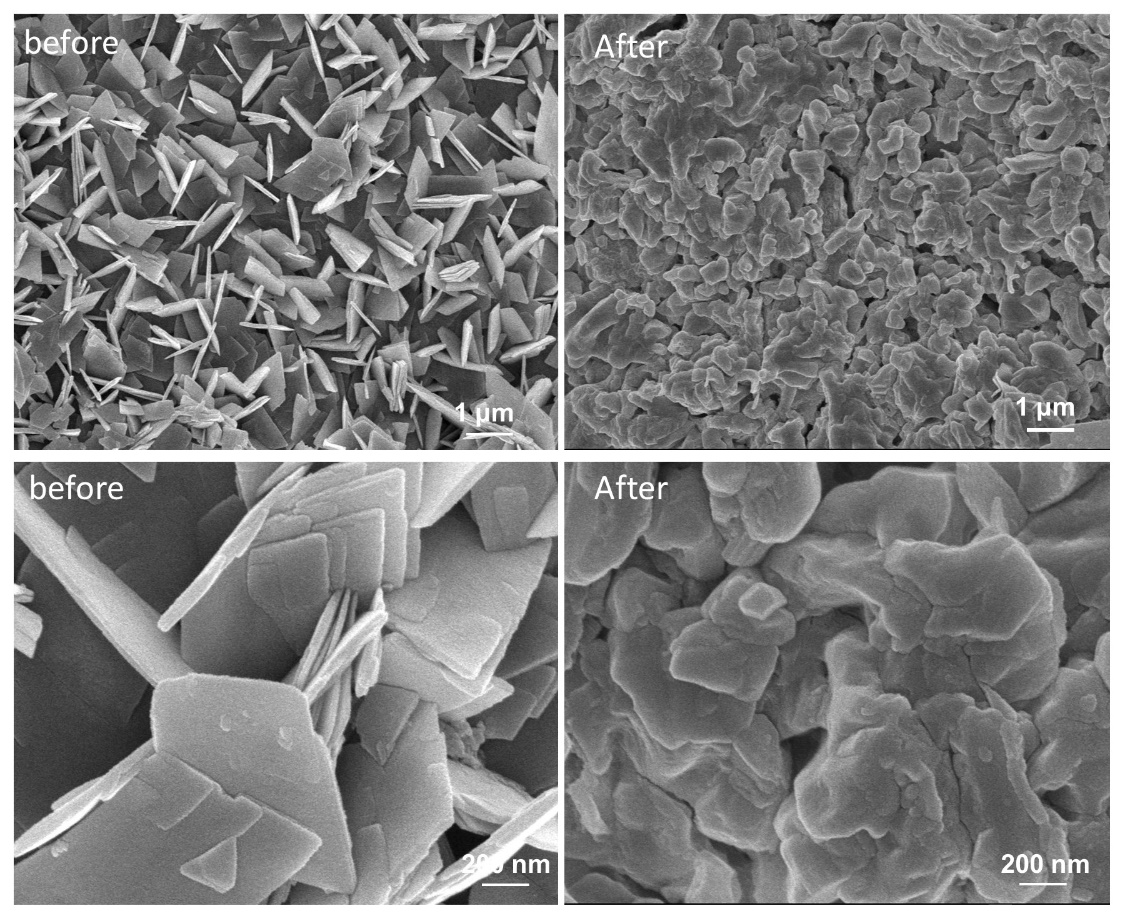


**Figure S9. SEM images of NiCo-AB MOFs before and after the stability test.** The morphology of the catalyst has not changed significantly. The long cycle blunts the morphology of the surface of the catalyst, but it is still uniform.


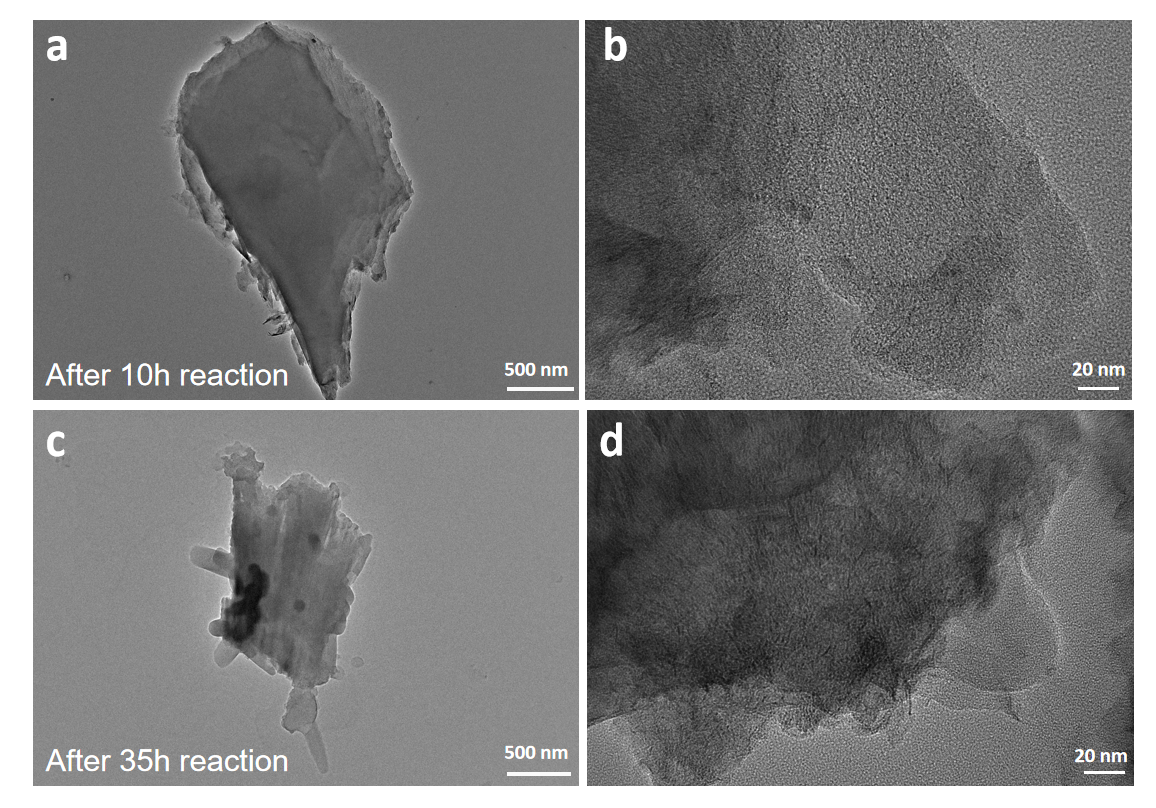


**Figure S10.** **TEM images of NiCo-AB MOFs after cyclic stability testing** (a for TEM after 10 hours of reaction, b for corresponding magnified image; c for TEM after 35 hours of reaction, d for corresponding magnified image).


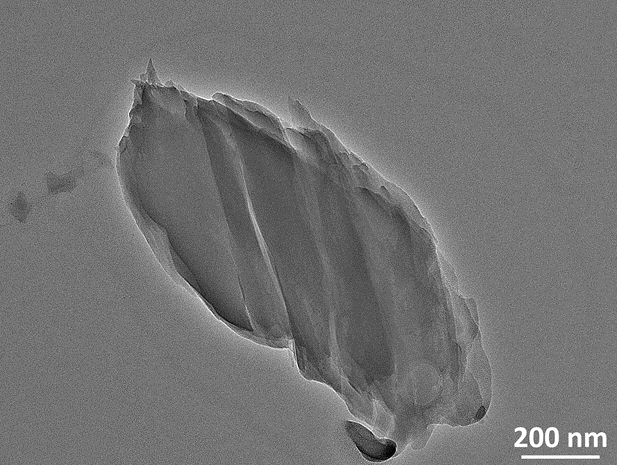


**Figure S11.** **TEM image of pristine NiCo-AB MOFs**.


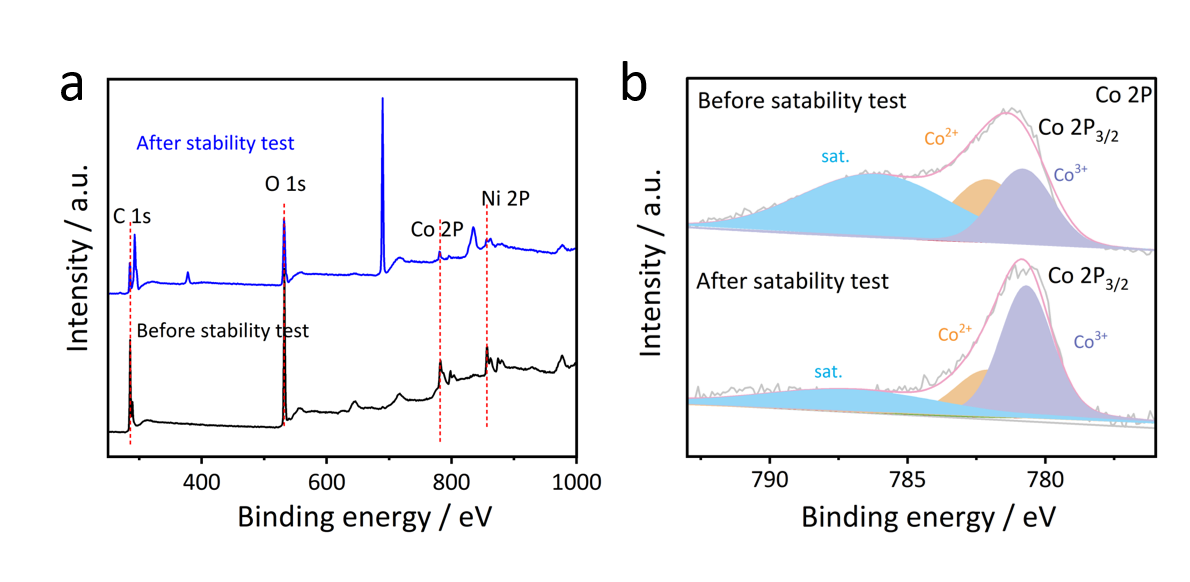
**Figure S12.** **(a) XPS spectra of NiCo-AB MOFs before and after stability testing; (b) The comparison of the peaks of the active center Co before and after the stability test.**


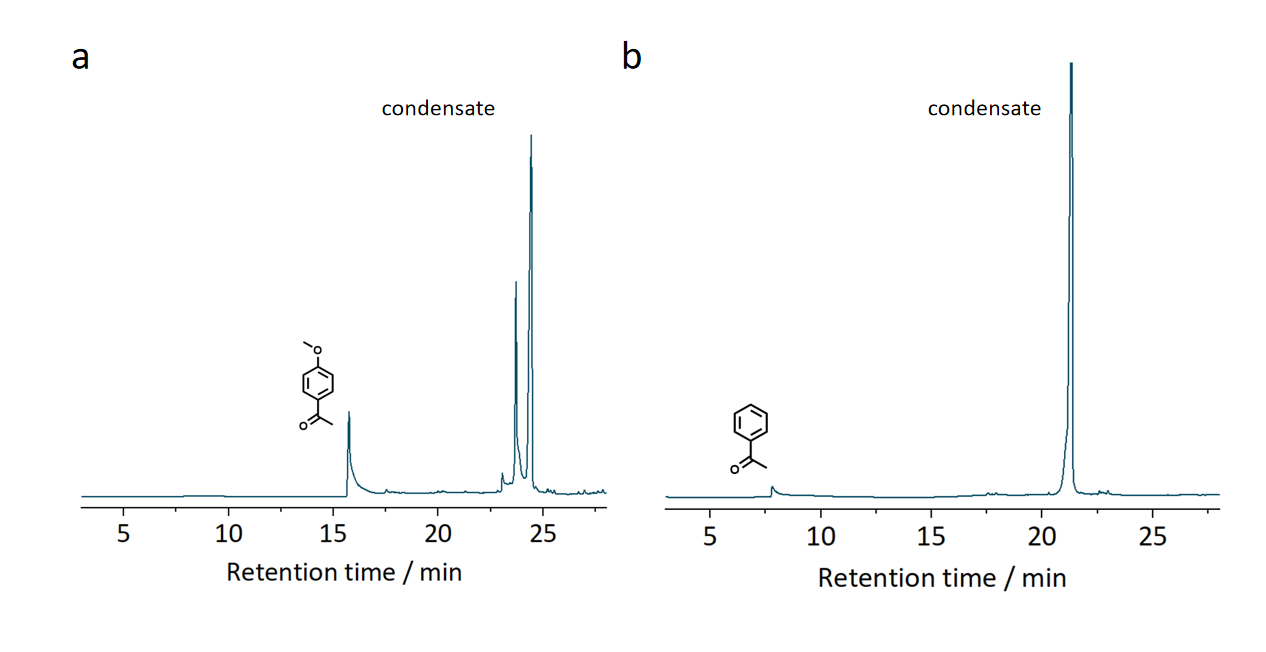


**Figure S13. Conversion of acetophenone with different functional group substituents:** 0.02 mmol of ketones were used as reaction substrates. Acetophenone structures without substituents or with only methoxy substituents undergo severe condensation after the reaction catalyzed by NiCo-AB MOFs, the substrates were all condensed severely. This is strong evidence that the yield of ketone products is lower than that of phenolic products when the dimer model is subjected to bond breaking.


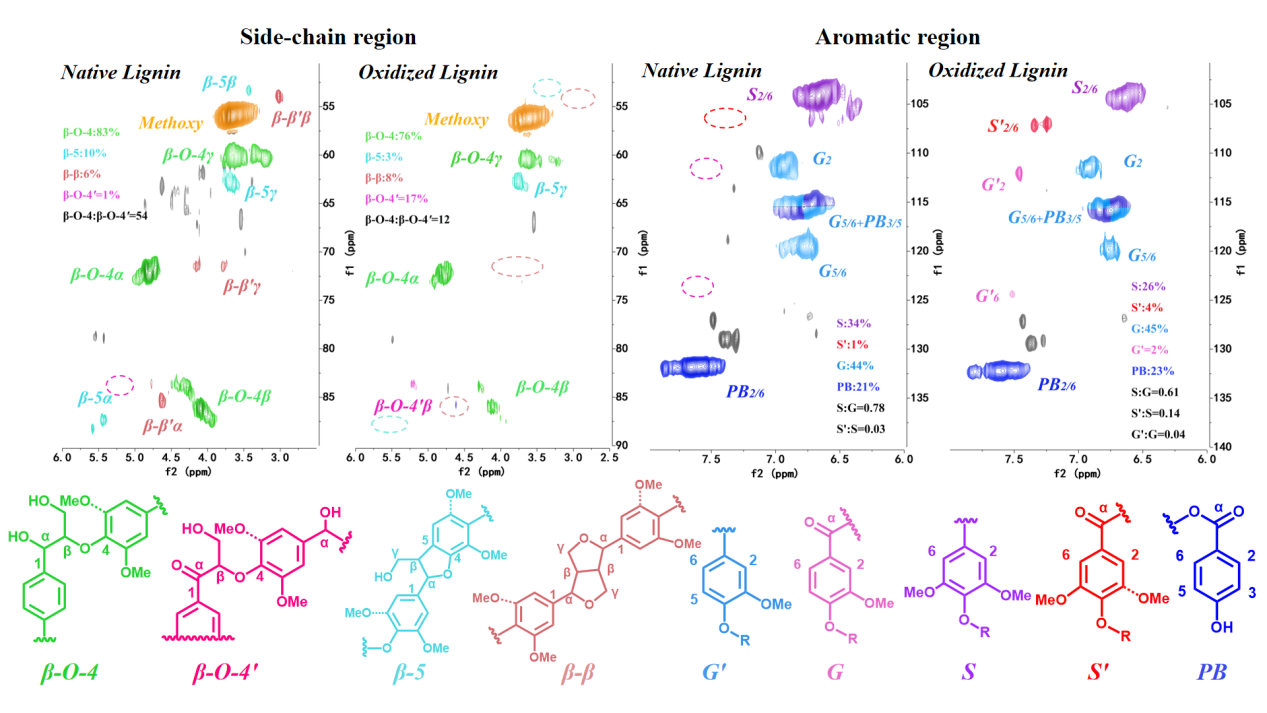


**Figure S14**. **Partial 2D HSQC NMR spectra of poplar lignin before and after oxidation.**


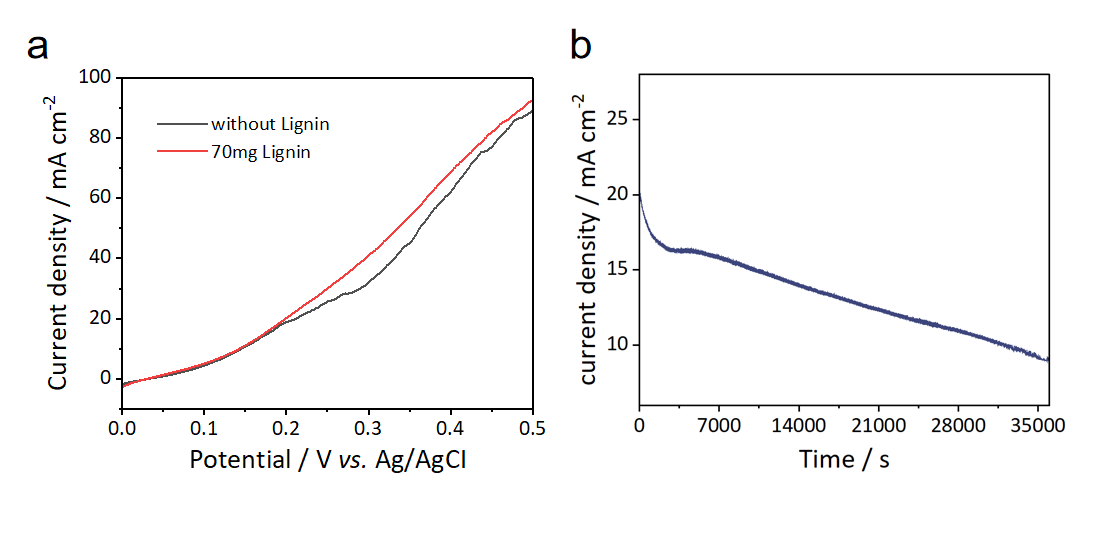


**Figure S15. LSV (a) and i-t curve (b) of electrocatalytic depolymerization of poplar lignin.**


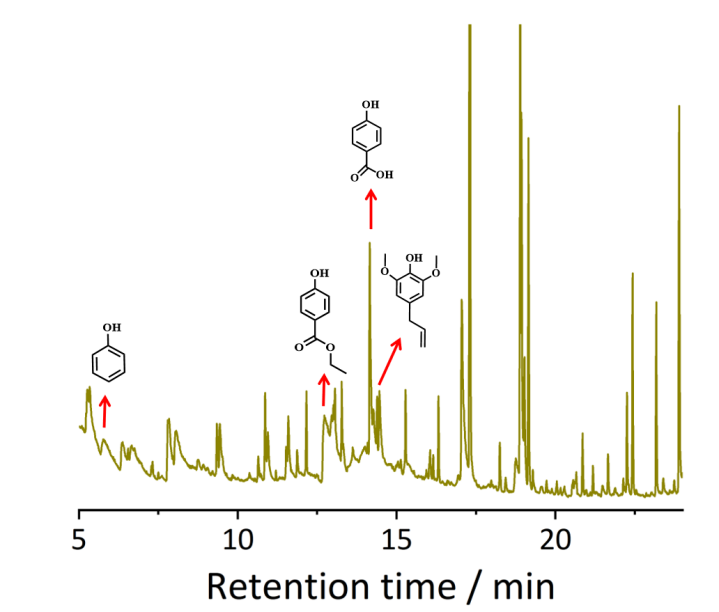


**Figure S16. GC-MS of electrocatalytic depolymerization of unoxidized poplar lignin.** Lignin without pre-oxidation contains a small amount of Cα-ketone, which leads to a reduced product type and low yield.

**Table S1.** ICP-AES analysis and N_2_ adsorption-desorption of catalysts before and after ligand doping.

| Catalyst | ICP-AES Analysis (wt%) | | S_BET_  (m^2^g^-1^) | V_pore_  (cm^3^g^-1^) | D_pore_  (nm) |
| --- | --- | --- | --- | --- | --- |
|  | Co | Ni |  |  |  |
| NiCo-A MOFs  NiCo-AB MOFs | 0.816  1.343 | 0.735  1.264 | 30.8  40.8 | 7.07  11.0 | 13.0  8.3 |

**Table S2.** Comparison of phenol yield from lignin model compounds on NiCo-AB MOFs with other catalysts.

| Catalyst | Reaction conditions | Yield of phenol / % | Conv. / % | Refs |
| --- | --- | --- | --- | --- |
| WE: NiCo-AB MOF | 0.34 V *vs.* Ag/ AgCl (30 mA/cm^2^), RT | 76.3 | 97 | This work |
| Pt | RT, air，t-BuOOH，  I=20mA | 27 | 93 | Ref.15 |
| Px-CoMo_4_-x/NF | 30 mA  RT | 48 | ＞99 | Ref.16 |
| Pt_1_/N-CNTs  nBu_4_NOH | 20 mA/cm^2^  RT | 56 | 97 | Ref.17 |
| TCP-Pd/CNT | RT, air, TBHP  15 mA | 18 | 99 | Ref.18 |
| Mo@NiCoOOH | nBu_4_NOH, TBHP | 13 | 93 | Ref.19 |
| ZIF-8-NH_2_@Bi/Bi_2_MoO_6_ | 300 W xenon lamp  (λ > 400 nm), RT | 57 | 93 | Ref.20 |
| [BMim ][NTf_2_] | air, 333K  UV | 50.4 | 95.1 | Ref.21 |
| [Ir(ppy)_2_(dtbpy)](PF_6_) | acetic acid, bluw LEDs | 79 | _ | Ref. 22 |
| KNb_6_–Cu/C_3_N_4_ | 80 ^o^C，O_2_ | 90 | 96 | Ref.23 |

**Table S3**. Comparison of performance of the lignin depolymerization on our NiCo-AB MOFs with other catalysts.

| Catalyst | Current/  potential | Temperature | Yields of phenol | Refs |
| --- | --- | --- | --- | --- |
| WE: NiCo-AB MOF | 0.34 V vs. Ag/ AgCl (30 mA/cm^2^) | RT | At least 13 kinds of products, including guaiacol, vanillin, p-hydroxybenzoic acid, eugenol, eugenoic acid, and others with the total yield higher than 12.48% | This work |
| Anode: Ni  Cathode: Pb/PbO_2_ | 30 mA/cm^2^ | 35°C | 8 products identified with yields up to 3.6 wt% | Ref. 24 |
| WE: Ni | 0.5V vs. Hg/HgO | RT | Vanillin and syringaldehyde with a combined maximum yield of 17.5% | Ref. 25 |
| WE:Ni/plate/wire/fleece/foam/foam stack | 8 A (3.17 mA/cm^2^） | RT | Vanillin, guaiacol, acetovanillone, syringaldehyde, et al. (all quantified did not exceed 0.5%) | Ref. 26 |
| NF@Co_3_S_4_/(α,β)-NiS | 1.414 V vs RHE | RT | Guaiacol, vanillin, acetovanillone and other kinds of products | Ref. 27 |
| Anode : Pt wire | 2.5 mA/cm^2^ | RT | The yield of EA soluble products was 36 wt% | Ref. 2 |
| Pt ,nBu_4_NOH | 20 mA/cm^2^ | RT | Obtaining 5% p-hydroxyacetophenone after β-O-4 polymer model reaction | Ref. 28 |
| Mo@NiCoOOH | nBu4NOH, TBHP,4V | RT | yield of up to 13 wt% of aromatic monomers | Ref.19 |

**References**

1. Rahimi, A., Azarpira, A., Kim, H., Ralph, J., & Stahl, S. S. Chemoselective metal-free aerobic alcohol oxidation in lignin. Journal of the American chemical society, *JACS* **2013**, 135, 6415-6418.
2. Fang, Z., Jackson, J. E., & Hegg, E. L., Mild, electroreductive lignin cleavage: Optimizing the depolymerization of authentic lignins. *ACS Sustainable Chem. Eng.* **2022**, 10, 7545-7552.
3. Li, Y., Yu, Y., Lou, Y., Zeng, S., Sun, Y., Liu, Y., Yu, H., Hydrogen‐Transfer Reductive Catalytic Fractionation of Lignocellulose: High Monomeric Yield with Switchable Selectivity. *Angew. Chem. Int. Ed.* **2023**, 135(32), e202307116
4. Kozlov, A. N. E. S. M., Viñes, F., & Illas, F., Electronic-structure-based chemical descriptors:(in) dependence on self-interaction and Hartree-Fock exchange. *Phys. Rev. B* **1996***,* 54, 29.
5. Kresse, G., Furthmüller, J. Efficiency of ab-initio total energy calculations for metals and semiconductors using a plane-wave basis set. *Comp Mater Sci* **1996**, 6, 15-50.
6. Hafner, J. Ab‐initio simulations of materials using VASP: Density‐functional theory and beyond. *J. Comput.* **2008**, 29, 2044-2078.
7. Blöchl, P. E., Projector augmented-wave method. *Phys. Rev. B* **1994***,* 50, 17953-17979.
8. Kresse, G., Joubert, D., *Phys. Rev. B* **1999**, 59, 1758-1775.
9. Hammer, B. H. L. B., Hansen, L. B., & Nørskov, J. K., Improved adsorption energetics within density-functional theory using revised Perdew-Burke-Ernzerhof functionals. *Phys. Rev. B* **1999**, 59, 7413-7421.
10. Frisch, M.J., et al., Gaussian 16, Revision A.03, Gaussian, Inc., Wallingford, CT, 2016.
11. Lee, C., W. Yang, and R.G. Parr, Development of the Colle-Salvetti correlation-energy formula into a functional of the electron density. *Phys. Rev. B* **1988**, 37, 785-789.
12. Becke, A.D., Density‐functional thermochemistry. III. The role of exact exchange. *J. Chem. Phys.* **1993**, 98, 5648-5652.
13. Cheng, W., Zhao, X., Su, H., Tang, F., Che, W., Zhang, H., & Liu, Q. Lattice-strained metal–organic-framework arrays for bifunctional oxygen electrocatalysis. *Nat. Energy* **2019**, 4, 115-122.
14. Yan, Y., Zhou, H., Xu, S. M., Yang, J., Hao, P., Cai, X., Duan, H. Electrocatalytic upcycling of biomass and plastic wastes to biodegradable polymer monomers and hydrogen fuel at high current densities. *JACS* **2023**, 145, 6144-6155.
15. Ma, L., Zhou, H., Kong, X., Li, Z., Duan, H. An electrocatalytic strategy for C-C bond cleavage in lignin model compounds and lignin under ambient conditions.**2021** *ACS Sustain. Chem. Eng.*, 9, 1932-1940.
16. Qi, Y., Liu, B., Qiu, X., Zeng, X., Luo, Z., Wu, W., Qin, Y. . Simultaneous Oxidative Cleavage of Lignin and Reduction of Furfural via Efficient Electrocatalysis by P‐Doped CoMoO_4_. *Adv. Mater.* **2023**, 35, 2208284.
17. Cui, T., Ma, L., Wang, S., Ye, C., Liang, X., Zhang, Z., Li, Y. Atomically dispersed Pt-N3C1 sites enabling efficient and selective electrocatalytic C-C bond cleavage in lignin models under ambient conditions. *JACS* **2020**, 143, 9429-9439.
18. Ren, P., Shi, L., Kan, Z., Bai, J., Liu, Y., Yang, S., Liu, S. Molecular palladium catalyst enabling efficient electrochemical C-C bond cleavage within lignin model compounds. *Catal. Sci. Technol.*, 2024, 14, 973–979
19. Xu, J., Meng, J., Hu, Y., Liu, Y., Lou, Y., Bai, W., Wang, S. Electrocatalytic Lignin Valorization into Aromatic Products via Oxidative Cleavage of Cα-Cβ Bonds. *Research*, 2023, 6, 0288.
20. Dai, D., Qiu, J., Xia, G., Tang, Y., & Yao, J. Defect Engineering Promoted Photocatalysis for Lignin Depolymerization: Performance and Mechanism Insight. *ACS Catal* **2023**, 13, 14987-14995.
21. Kang, Y., Yao, X., Yang, Y., Xu, J., Zhou, Q., Zhang, S. Metal-free and mild photo-thermal synergism in ionic liquids for lignin Cα-Cβ bond cleavage to provide aldehydes. *Green Chem*, **2021**, 23, 5524-5534.
22. Zhu, Q., & Nocera, D. G. Catalytic C(β)-O bond cleavage of lignin in a one-step reaction enabled by a spin-center shift. *ACS Catal*, 2021, 11, 14181-14187.
23. Li, J., Li, Z., Dong, J., Fang, R., Chi, Y., & Hu, C. Hexaniobate as a Recyclable Solid Base Catalyst to Activate C-H Bonds in Lignin Linkage Boosting the Production of Aromatic Monomers. *ACS Catal.*, **2023**, 13(8), 5272-5284.
24. Lan, C., Fan, H., Shang, Y., Shen, D., & Li, G., Electrochemically catalyzed conversion of cornstalk lignin to aromatic compounds: an integrated process of anodic oxidation of a Pb/PbO_2_ electrode and hydrogenation of a nickel cathode in sodium hydroxide solution. *Sustain. Energ Fuels* **2020**, 41828-1836.
25. Yan, K., Zhang, Y., Tu, M., & Sun, Y. Electrocatalytic valorization of organosolv lignin utilizing a nickel-based electrocatalyst. *Energy Fue* **2020**, 34, 12703-12709.
26. Stiefel, S., Schmitz, A., Peters, J., Di Marino, D., & Wessling, M., An integrated electrochemical process to convert lignin to value-added products under mild conditions. *Green Chem.* **2016** 18, 4999-5007.
27. Du, X., Zhang, H., Sullivan, K. P., Gogoi, P., Deng, Y. Electrochemical lignin conversion. *ChemSusChem* **2020,** 13, 4318-4343.
28. Ma, L., Zhou, H., Kong, X., Li, Z., & Duan, H. An electrocatalytic strategy for C-C bond cleavage in lignin model compounds and lignin under ambient conditions. *ACS Sustain. Chem. Eng.* **2021**, 9, 1932-1940.
